# Dorsal Raphe Nucleus Down-Regulates Medial Prefrontal Cortex during Experience of Flow

**DOI:** 10.3389/fnbeh.2016.00169

**Published:** 2016-09-05

**Authors:** Martin Ulrich, Johannes Keller, Georg Grön

**Affiliations:** ^1^Department of Psychiatry, University of Ulm, UlmGermany; ^2^Department of Psychology and Education, University of Ulm, UlmGermany

**Keywords:** flow experience, dynamic causal modeling, effective connectivity, dorsal raphe nucleus, medial prefrontal cortex, amygdala, midbrain, functional magnetic resonance imaging

## Abstract

Previous neuroimaging studies have suggested that the experience of flow aligns with a relative increase in activation of the dorsal raphe nucleus (DRN), and relative activation decreases of the medial prefrontal cortex (MPFC) and of the amygdala (AMY). In the present study, Dynamic Causal Modeling (DCM) was used to explore effective connectivity between those brain regions. To test our hypothesis that the DRN causally down-regulates activity of the MPFC and/or of the AMY, 23 healthy male students solved mental arithmetic tasks of varying difficulty during functional magnetic resonance imaging. A “flow” condition, with task demands automatically balanced with participants’ skill level, was compared with conditions of “boredom” and “overload”. DCM models were constructed modeling full reciprocal endogenous connections between the DRN, the MPFC, the AMY, and the calcarine. The calcarine was included to allow sensory input to enter the system. Experimental conditions were modeled as exerting modulatory effects on various possible connections between the DRN, the MPFC, and the AMY, but not on self-inhibitory connections, yielding a total of 64 alternative DCM models. Model space was partitioned into eight families based on commonalities in the arrangement of the modulatory effects. Random effects Bayesian Model Selection (BMS) was applied to identify a possible winning family (and model). Although BMS revealed a clear winning family, an outstanding winning model could not be identified. Therefore, Bayesian Model Averaging was performed over models within the winning family to obtain representative DCM parameters for subsequent analyses to test our hypothesis. In line with our expectations, Bayesian averaged parameters revealed stronger down-regulatory influence of the DRN on the MPFC when participants experienced flow relative to control conditions. In addition, these condition-dependent modulatory effects significantly predicted participants’ experienced degree of flow. The AMY was down-regulated irrespective of condition. The present results suggest a causal role for the DRN in modulating the MPFC, contributing to the experience of flow.

## Introduction

“Flow” can be experienced when individuals engage in a challenging task or activity with demands optimally balanced to the individual skill level. Among other features, effortless involvement, a deep sense of control, and suspension of self-reflective thoughts are hallmarks of the experience of flow (Csikszentmihalyi, 1975, 1990). From a conceptual, more teleological perspective and acknowledging the defining features fostering the positive experience of flow, one can also argue that flow *per se* may qualify as a very potent psychological mechanism in motivating major individual achievement in functional domains that permit the experience of flow. It is easy to imagine that some important cultural advances (e.g., in the field of music, literature, painting, or science) have been achieved by individuals whose strong engagement was driven by the experience of flow, since, in order to maintain the positive experience of flow, it is necessary to steadily increase task demands once a certain level of competence has been reached. This self-reinforcing feature inherent in the experience of flow therefore appears to represent a cardinal mechanism of intrinsic motivation which should be added to the conceptual background whenever discussing this specific phenomenon.

In recent years, brain imaging studies have begun to explore the neural correlates of flow (de Manzano et al., 2013; Ulrich et al., 2014, 2016; Harmat et al., 2015). In two of those studies (Ulrich et al., 2014, 2016), mental arithmetic tasks were employed to gain precise control over task difficulty: Two conditions were meant to bore or to overwhelm participants by presenting very simple or very difficult calculations, respectively. In another condition, task difficulty was continuously and automatically adapted to individuals’ level of mental arithmetic skills. By balancing demands and skills, this condition was supposed to induce flow experience, mirrored by specific brain activation. An early study (Ulrich et al., 2014) employing magnetic resonance (MR) perfusion imaging indicated that flow seems to be associated with two basic patterns of neural activation: Brain regions reported to perform task-general computations (“multiple-demand system”; Duncan, 2010) such as the inferior frontal gyrus and the anterior insula demonstrated higher activation under flow (F) compared with boredom (B) and overload (O). Conversely, a different set of regions that can be described as a subset of the “default-mode network” (Gusnard et al., 2001; Raichle et al., 2001), including the medial prefrontal cortex (MPFC), lateral temporo-parietal cortex, and the amygdala (AMY), showed relative decreases in neural activation during the flow experience. More recent work has largely replicated those findings utilizing a typical functional magnetic resonance imaging (fMRI) block-design and measuring the blood oxygenation level-dependent (BOLD) signal (Ulrich et al., 2016). Due to the relatively higher sensitivity of BOLD imaging compared to perfusion imaging (Yang et al., 2005; Liu and Brown, 2007; Wang et al., 2011), that study could also confirm the dorsal raphe nucleus (DRN) to play a significant role in mediating flow experience. Neural activation of the DRN was markedly increased during flow relative to control conditions, which had already been found in our perfusion imaging study (Ulrich et al., 2014), but could not be reported since that differential effect did not surpass statistical thresholding.

Among the various regions with differentially increasing and decreasing neural activity during flow relative to control conditions (Ulrich et al., 2014, 2016), the MPFC and the AMY were the only structures where relative decreases in neural activity during flow repeatedly correlated with the degree of subjective flow experience. Both brain regions have already been reported to mediate ruminative self-reflection (Gusnard et al., 2001; Northoff et al., 2006; D’Argembeau et al., 2007; Jenkins and Mitchell, 2011; Philippi et al., 2012) and emotional arousal (Hamann et al., 2002; McGaugh, 2004; Lewis et al., 2007; Colibazzi et al., 2010), which represent crucial features in the definition of flow as a psychological construct (Csikszentmihalyi, 1990; Peifer, 2012).

From that background, with decreasing activity of the MPFC and AMY and increasing activity of the DRN during flow, in the present study, we investigated whether and how these brain regions would effectively interact during flow relative to control conditions. Inclusion of the DRN into this network was further motivated by previous work showing that strong serotonergic projections exist from the DRN to the MPFC and AMY (Bobillier et al., 1976; Ma et al., 1991; Rainnie, 1999; Hajós et al., 2003; Puig et al., 2005; Jiang et al., 2009; Hahn et al., 2012), and research in depressive patients has demonstrated that pharmacologically induced increases in serotonergic tone is efficient in decreasing MPFC and AMY activity, concomitantly reducing symptoms of rumination and accompanying arousal (Drevets, 2000; Mayberg et al., 2000; Brody et al., 2001; Drevets et al., 2002; Sheline et al., 2009; Cooney et al., 2010; Nejad et al., 2013; van de Ven et al., 2013).

In the present investigation, we therefore re-analyzed our previous fMRI data (Ulrich et al., 2016) using Dynamic Causal Modeling (DCM; Friston et al., 2003) to estimate effective connectivity between the DRN, the MPFC, the AMY, and the calcarine (through which visual input was assumed to enter the brain). Assuming full reciprocal endogenous connections between all four brain regions, 64 alternative models were set up differing only in the specific connections assumed to be modulated by the experimental conditions of boredom, flow, and overload. Based on commonalities in the specific arrangement of the modulatory effects, the models were grouped into eight families. Families/models were compared using random effects Bayesian Model Selection (BMS; Penny et al., 2010). To test the hypothesis that the DRN down-regulates the MPFC and/or the AMY strongest under flow relative to control conditions, representative parameters describing the condition-specific modulatory effects on the DRN-to-MPFC and DRN-to-AMY connections were extracted for all participants and then tested for significant differences between conditions. Additional correlation analyses were performed to test whether the modulatory effects on the MPFC and/or the AMY robustly predicted participants’ “flow index”, a condensed measure of the subjectively experienced degree of flow (Ulrich et al., 2014, 2016).

## Materials and Methods

### Participants

Participants were the same as those mentioned in our previous research report (Ulrich et al., 2016). Twenty-three healthy right-handed male students with a mean age of 24 years (standard deviation: 2.7 years). Written informed consent was obtained prior to the experiment. The study was in accordance with the ethics committee at the University of Ulm and with the Declaration of Helsinki.

### Experimental Design

The experimental procedure is identical to that reported in our previous work (Ulrich et al., 2016). In brief, the experiment consisted of 27 task blocks during which participants had to solve mental arithmetic tasks consisting of two or more summands that were visually presented on MRI compatible video goggles (VisuaStim Digital, Resonance Technology Inc., Northridge, CA, USA). Subjects had to sum the numbers in their mind and to enter the result as accurately and fast as possible (within a maximum period of 10 s) using an on-screen keyboard controlled by a trackball (NAtA TECHNOLOGIES, Coquitlam, BC, Canada). Upon submission of the result or when the time limit of 10 s was exceeded, there was a short 1 s break indicated by the string “xxx + x”. Then the next calculation item appeared. Math expressions and intermitting breaks were presented during the entire block length of 30 s.

The blocks differed in task difficulty which was modulated by the number of summands per math expression and by whether the last summand had one digit versus two digits (for details, see Ulrich et al., 2016). There were nine blocks of a “boredom” condition which demanded very little from participants (e.g., “102 + 5”). Further nine blocks represented the “overload” condition, i.e., task difficulty exceeded subjects’ pre-experimentally determined skill level. In the remaining nine blocks, the difficulty of a given math expression at hand was automatically adapted to participants’ performance on the previous calculation. By continuously balancing task demands with the individual’s level of mental arithmetic skill, we aimed at inducing a flow experience (“flow” condition).

There were two fixed block orders counterbalanced across participants. Between task blocks of either condition as well as at the beginning and at the end of the experiment, there were resting blocks (30 s) where participants were asked to fixate a black cross on white background.

The software generating and presenting the math tasks, analyzing the results, and adjusting task difficulty was programmed in Scala version 2.9.01 and run in Java Runtime Environment version 6.0.05 on a standard PC with Windows XP Professional version 2002 Service Pack 3.

After the experiment, we informed participants about the presence of three basic task conditions varying in the level of difficulty (labeled “easy”, “moderate”, and “difficult” for boredom, flow, and overload, respectively) and then asked them to retrospectively respond to “some questions on their subjective experiences” related to each of the three conditions (in the order B–F–O). Three statements were presented to participants, assessing core elements of the flow experience: “I would love to solve math calculations of that kind again”, “Task demands were well matched to my ability”, and “I was thrilled”. Participants’ responses on Likert scales could range from 1 (“I do not agree at all”) to 7 (“I completely agree”). From the responses given, the “flow index”, an index of the individually experienced level of flow (Ulrich et al., 2014, 2016), was obtained by computing a score based on the formula “-B + 2F - O” from each item’s rating scores and summing up these scores across the three items.

### MRI Data Acquisition

Functional images were acquired on a 3 Tesla magnetic resonance scanner (MAGNETOM Allegra, Siemens AG, Erlangen, Germany) in combination with a single channel transmit/receive head coil (RAPID Biomedical GmbH, Rimpar, Germany). During the experiment, an echo-planar pulse sequence (EPI) was applied to measure the T2*-weighted BOLD signal. The following parameters were used: repetition time = 2000 ms, echo time = 35 ms, bandwidth = 3396 Hz/Px, flip angle = 90°, field of view = 228 mm, matrix size = 64 × 64, number of slices = 33, slice thickness = 3.0 mm, interslice gap = 0.6 mm, isotropic voxel size of 3.6 mm^3^. Ascending slice acquisition was parallel to the anterior/posterior commissure line. Scan time was about 27 min, corresponding to 825 EPI volumes. To obtain a high resolution T1-weighted structural image for later coregistration purposes, a magnetization prepared rapid acquisition gradient echo sequence was employed (repetition time = 2500 ms, echo time = 4.57 ms, inversion time = 1100 ms, bandwidth = 130 Hz/Px, flip angle = 12°, field of view = 256 mm, matrix size = 256 × 256, voxel volume = 1 mm^3^, slice orientation: sagittal; scan time about 9 min).

### Dynamic Causal Modeling

As outlined in the Introduction, we hypothesized that relatively increased activation of the DRN under flow down-regulates activation of the MPFC and/or of the AMY. This hypothesis was tested by applying DCM to our previous fMRI data set (Ulrich et al., 2016). We used the most recent version of Statistical Parametric Mapping 12 (SPM12 r6685, Wellcome Department of Cognitive Neurology, London, UK) to perform first and second level analyses of brain activation as well as DCM (DCM12) on the already preprocessed (i.e., realigned, normalized, and smoothed) EPI images. Explicitly, slice timing correction had not been performed, but different slice acquisition times were accounted for during DCM model construction (see below).

The DCM approach requires a design matrix, with the regressors providing the inputs for the model, as well as the BOLD signal time courses of the brain regions whose neural dynamics are to be modeled. The single-subject design matrix was set up by entering the onsets and durations (30 s) of the task blocks for boredom, flow, and overload, to form three separate regressors. As conditions of no interest the spatial realignment parameters were included. Resulting box-car functions were convolved with the canonical hemodynamic response function. Low-frequency scanner drifts were removed by high-pass filtering (cutoff: 128 s). To account for temporally correlated residual errors, an autoregression model of polynomial order 1 was used. After model estimation, contrast images representing activation under experimental conditions B, F, and O relative to implicit baseline were obtained from all participants and subjected to a random effects analysis (flexible factorial design) with factors *experimental condition* and *subject*. The group analysis was used to define volumes of interest (VOI) from which single-subject time courses were extracted.

Based on our hypothesis, models were set up to include four brain regions: (1) an early visual region through which sensory input would enter the system, (2) the DRN, (3) the MPFC, and (4) the AMY.

To identify an early visual region, directed t-contrasts testing for greater activation under task conditions (B, F, and O) relative to baseline were conjoined and the statistical parametric map was thresholded at *p* < 0.050, family-wise error-corrected at the voxel level. The maximum of this conjoined contrast was located in the left calcarine (Montreal Neurological Institute [MNI] coordinates: [-14, -92, -12]), and was defined as VOI 1.

Definition of the DRN was driven functionally and anatomically. First we identified the peak voxel by testing for significant positive flow-related activation using the contrast [-B + 2F - O] (see Ulrich et al., 2016), thresholded at *p* < 0.001 at the voxel level and family wise error corrected for multiple comparisons at the cluster level (*p* < 0.05). This peak voxel was located at MNI coordinates [-2, -26, -14]. To ensure, however, that this functionally derived position was in agreement with the DRN’s anatomy, we followed the method described by Kranz et al. (2012) to define the DRN on the basis of anatomical landmarks. As we recognized that the voxel [-2, -26, -14] fell onto the boundary of the anatomical DRN, we decided to instead use a neighboring but more posterior/superior voxel at [0, -28, -12] as VOI 2. That voxel was associated with the next highest effect size for the contrast above.

**FIGURE 1:**
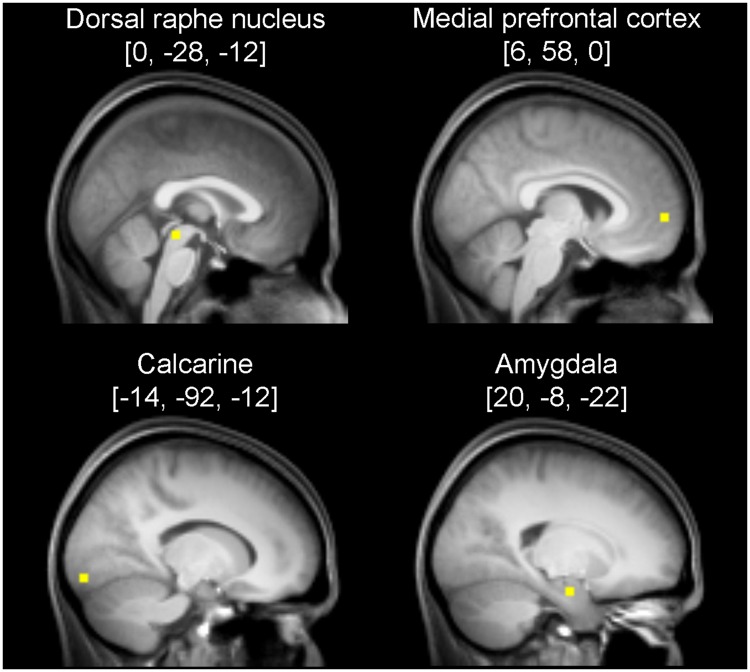
**Sagittal anatomical sections showing the volumes of interest (VOI) used for Dynamic Causal Modeling (DCM).** The VOIs consisted of one voxel each, but for better illustration, spheres with a radius of 3 mm are shown. Coordinates refer to Montreal Neurological Institute space.

Loci representing the MPFC and the AMY were yielded by applying the contrast [B - 2F + O] testing for relative deactivation under flow compared with boredom and overload. The statistical parametric map was again thresholded at *p* < 0.001 (voxel level) corrected for multiple comparisons at the cluster level (*p* < 0.05). Significant local maxima within the MPFC and the AMY, detected at [6, 58, 0] and [20, -8, -22], respectively, were defined as VOI 3 and VOI 4. VOIs contained one single voxel each. The location of the VOIs is shown in **Figure 1**.

Afterward, principal eigenvariate time-series were extracted at the single-subject level relying on the group level-defined VOI definitions described above. Data were adjusted for “effects of interest” using an appropriate F-contrast.

Several competing DCMs were constructed with the following commonalities for every participant: Direct input (represented by the DCM.c matrix) entered the system via the calcarine. Endogenous connections between the calcarine, the DRN, the MPFC, and the AMY were modeled such that all regions were fully reciprocally connected with each other and had self-inhibitory connections (i.e., all elements of the DCM.a matrix were set to 1). The models only differed in the connections upon which experimental context-specific modulatory effects were assumed to act, represented by the DCM.b matrix.

The experimental conditions were modeled to exert modulatory effects on the forward and/or the backward connections between one or more of the following brain regions: DRN-MPFC, DRN-AMY, and MPFC-AMY. Modulatory effects on self-inhibitory connections and on connections involving the calcarine were not modeled. Moreover, to facilitate comparability between experimental conditions, for any given DCM the modulatory effects of B, F, and O were modeled to affect the same connections, that is, the three dimensions of the DCM.b matrix representing B, F, and O, respectively, were identical. This yielded 2^6^ DCMs per participant. As summarized in **Table 1**, model space was partitioned into eight families based on commonalities in the specific arrangement of context-dependent modulatory effects, influencing either forward connections, backward connections, or both, between DRN and MPFC, and/or DRN and AMY, and/or MPFC and AMY. Detailed information on matrices DCM.a, DCM.b, and DCM.c of all 64 models is presented as Supplementary Material.

**Table 1:** Partitioning of model space into eight families based on commonalities in the arrangement of context (“boredom”, “flow”, “overload”)-dependent modulatory effects on connections between DRN and MPFC, and/or DRN and AMY, and/or MPFC and AMY.

	DRN-MPFC	DRN-AMY	MPFC-AMY
Family 1	✓	✓	✓
Family 2	✓	✓	
Family 3	✓		✓
Family 4		✓	✓
Family 5	✓		
Family 6		✓	
Family 7			✓
Family 8			

*A check mark indicates the presence of modulatory effects either on the forward connection, the backward connection, or both, for a given pair of brain regions. Abbreviations: AMY, amygdala; DRN, dorsal raphe nucleus; MPFC, medial prefrontal cortex.*

Dynamic Causal Models were set up with one state per region, with bilinear modulatory effects, and without mean-centering of inputs. Because locations of the VOIs corresponded to different slices during individual MRI acquisition, the group level-derived MNI coordinates of the VOIs were inversely normalized to participants’ individual EPI coordinate space reflecting the original image orientation during MRI acquisition before preprocessing. For every participant, the pre-normalized *z*-coordinates of the VOIs were used to compute the respective acquisition times (in seconds) which were then entered into the parameter “DCM.delays” (cf. Kiebel et al., 2007).

Models were estimated for all participants and compared using random effects BMS (Penny et al., 2010) to find the family (and the model within the winning family) associated with the highest evidence.

## Results

**FIGURE 2:**
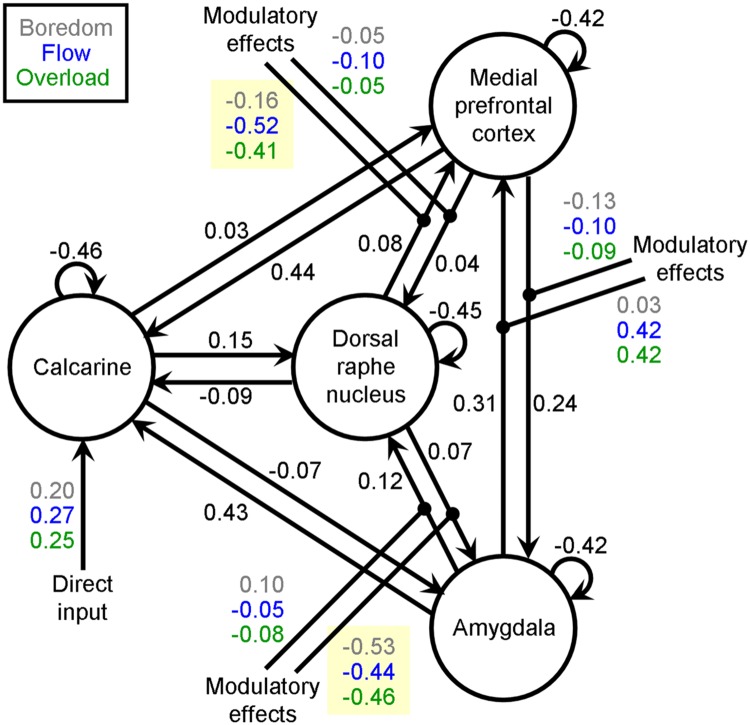
**Average parameters (in Hz) over models and participants for family 1 computed by Bayesian Model Averaging after identifying family 1 as the winning family using random effects Bayesian Model Selection.** Parameters in black refer to endogenous connection strengths. Direct input and modulatory effects on connections are coded in different colors to represent the experimental conditions boredom (gray), flow (blue), and overload (green). Parameters highlighted in yellow were subjected to repeated measures ANOVAs to test our hypothesis that the dorsal raphe nucleus (DRN) exerted down-regulating influence on activity of the medial prefrontal cortex and/or the amygdala (AMY) in a condition-dependent manner. Whereas the modulatory effects on the medial prefrontal cortex differed significantly between boredom, flow, and overload [*F*(2,44) = 4.37, *p* = 0.019], this was not the case for the AMY [*F*(2,44) = 0.16, *p* = 0.853)]

Random effects BMS revealed family 1 as the winning family with a family exceedance probability of 83.7%. However, there was no clear winning model within family 1. All models had exceedance probabilities lower than 31.2%. Therefore, Bayesian Model Averaging (BMA, Penny et al., 2010) was applied to obtain Bayesian average parameters over models and participants for family 1. For computational efficiency, BMA implemented in DCM12 uses an Occam’s window approach: per participant only models with a probability higher than the minimal posterior odds ratio (1/20) are considered. Subject-specific parameter averaging is then performed by weighting parameters by the posterior probability of each model included. In the present study, the average number of models in Occam’s window per participant was 2.7 (standard deviation: 1.7). The group-averaged BMA parameters are presented in **Figure 2**. The main finding with regard to our hypothesis was that down-modulatory effects on the forward connection from the DRN to the MPFC was strongest during the experience of flow compared with control conditions, and is reflected by a more negative rate of change of MPFC activity under flow (-0.52 Hz) than under boredom (-0.16 Hz) and overload (-0.41 Hz). To test for significant differences between conditions, subject-specific parameters (from BMS.DCM.rfx.bma.mEps) underlying the group mean were passed to a repeated measures analysis of variance (ANOVA) with *experimental condition* constituting the within-subject factor. There was a significant overall effect of *experimental condition* [*F*(2,44) = 4.37, *p* = 0.019], and *post hoc* Newman–Keuls tests indicated a significant difference between boredom and flow (*p* = 0.017) but not between overload and flow (*p* = 0.417).

The analysis was repeated for effective connectivity between DRN and AMY. We found no evidence of a significant context-dependent modulation of AMY activation exerted by the DRN, however, [*F*(2,44) = 0.16, *p* = 0.853].

**FIGURE 3:**
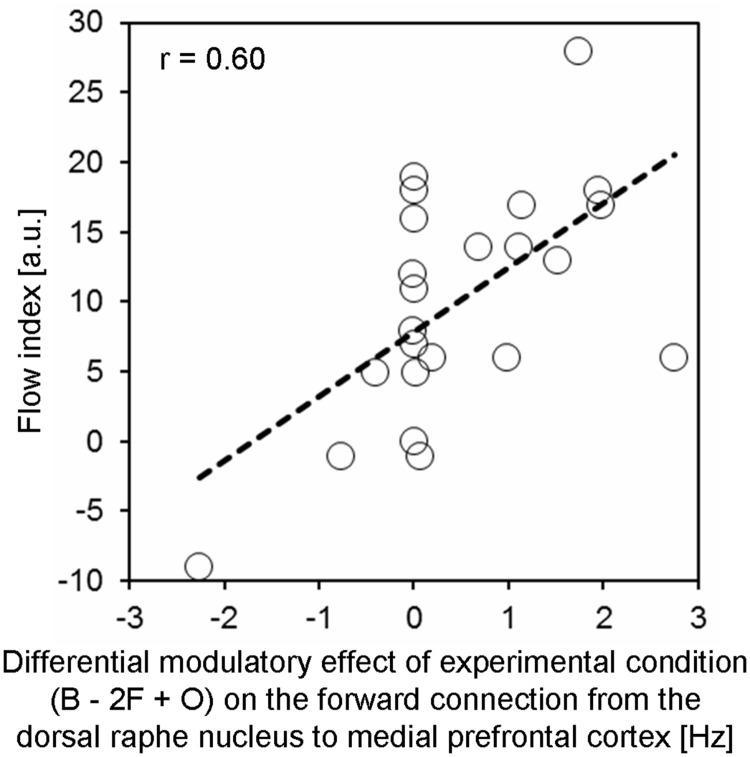
**The differential modulatory effect between experimental conditions on the forward connection from the DRN to the medial prefrontal cortex significantly (*r* = 0.60, *p* = 0.001) predicted participants’ flow index (arbitrary unit, a.u.).** The term “differential modulatory effect” refers to the difference in the rates of change of MPFC activity (caused by the DRN) between experimental conditions, calculated using the formula “B - 2F + O”. The respective subject-specific parameters were derived from the BMS.DCM.rfx.bma.mEps structure after Bayesian Model Averaging over models of the winning family (i.e., family 1; see Results section for details).

To test whether the modulatory effects acting on the MPFC were relevant for subjective experiences, an additional correlation analysis was performed. We expected that the more negative the DRN’s down-regulating effect on activity of the MPFC under flow relative to control conditions, the higher the flow index which reflected the subjectively experienced degree of flow. Per each subject the difference “B - 2F + O” in Bayesian averaged effective connectivity parameters was computed for the modulatory effect on the forward connection from the DRN to the MPFC, and then correlated with the flow index across participants. This correlation was highly significant [*r* = 0.60, *t*(22) = 3.41, *p* = 0.001, one-sided], meaning that context-dependent down-regulation robustly predicted participants’ flow index (**Figure 3**).

## Discussion

In the present investigation, 23 healthy male students underwent fMRI while solving mental arithmetic tasks that were optimally balanced with individual skill levels to induce experience of flow (see also Ulrich et al., 2016). DCM was applied to this fMRI data set to explore neural interactions between three selected candidate brain regions most likely mediating the flow experience. We found evidence supporting the hypothesis that the DRN down-regulates neural activation of the MPFC during the experience of flow. Individual rates of change of MPFC activity under flow relative to control conditions significantly associated with individual subjective reports of experiencing flow.

Present results provide initial corroboration of the hypothesis that the DRN may drive the emergence of flow experience. The DRN is the origin of strong serotonergic projections which have been suggested to decrease activity of the MPFC and of the AMY (Hajós et al., 2003; Puig et al., 2005; Jiang et al., 2009; Hahn et al., 2012). This is, for instance, supported by studies showing that selective serotonin reuptake inhibitors attenuate MPFC hyperactivity, concomitant with ameliorated depressive symptoms of negatively valenced rumination (e.g., Drevets, 2000; Mayberg et al., 2000; Brody et al., 2001; Drevets et al., 2002; Sheline et al., 2009; Cooney et al., 2010; Nejad et al., 2013; van de Ven et al., 2013).

Ruminative responding is not necessarily a cardinal feature of depressive syndromes, but also appears in healthy controls at different levels, reflecting self-referential processing (Gusnard et al., 2001; Northoff et al., 2006; D’Argembeau et al., 2007; Jenkins and Mitchell, 2011; Philippi et al., 2012) associated with negative affect (reviewed in Nejad et al., 2013; Hamilton et al., 2015). Resting state studies have repeatedly shown that the MPFC as a part of the default-mode network is involved in mediating ruminative self-reflection which is why decreased MPFC activity under flow may reflect a crucial neurophysiological signature underlying absence of self-reflective thoughts (Csikszentmihalyi, 1990; Peifer, 2012), mainly driven by the DRN.

While the relative decrease in AMY activity under flow (Ulrich et al., 2014, 2016) is in good agreement with its role in emotional arousal (Hamann et al., 2002; McGaugh, 2004; Lewis et al., 2007; Colibazzi et al., 2010) and with reports that AMY deactivation aligns with positive emotions (Morris et al., 1996; Whalen et al., 1998; Kim et al., 2004; Straube et al., 2008), one putative idea that decreased AMY activity is driven by MPFC’s rate of change (e.g., Phillips et al., 2008; Rive et al., 2013; Etkin et al., 2015), could not be supported by present DCM results (**Figure 2**). Our alternative hypothesis that direct DRN influences might have downregulated activity of the AMY most strongly during flow is also not supported by the present data, since negative effective connectivity between the DRN and the AMY was of comparable magnitude under all experimental conditions.

One aspect that appears more crucial than others for future work is the presently observed absence of a clear winning model during BMS, which can be considered as an indicator of too much interindividual variation. One putative factor that may be of relevance in this context is the time interval during which experimentally induced flow was studied. While this interval has proved useful in contrasting average neural activity between the different conditions of boredom, flow, and overload at the group level, and is well suited to meet requirements of MR signal drift adjusting high-pass filtering, it is not unlikely that this interval is too short to activate the network of brain regions in sufficient timely reliability. As an alternative we would suggest to increase block durations to at least twice as much as present intervals, which on the other hand will motivate deeper evaluation of MR scanner-specific signal drift.

As another approach to further substantiate present findings, future research should attempt to experimentally modulate the putative influence of the DRN on MPFC activity. This could be achieved by modulation of the serotonergic system using serotonin agonists or antagonists, which would be expected to intensify or interrupt the experience of flow, respectively. Another possibility is offered by neurostimulation techniques, for instance, by transcranial direct current stimulation to enhance or to suppress MPFC activity. Once this initial mechanistic link between the DRN and MPFC has been further corroborated by additional empirical evidence as outlined above, more complex DCM models could be established and tested by including not only the DRN, the MPFC, and the AMY, but also other regions showing decreased activation under flow relative to control conditions, although signal decreases in these regions were not correlated with subjects’ individual flow experience which, so far, was the most crucial criterion to ascertain that those brain regions with relative signal changes during the flow condition do relate to its subjective experience.

The reason behind this brain-experience relationship is that flow *per se* is an amalgam of cognitive and affective processes whose different contributions to the observed, particularly the positive going relative signal changes cannot sufficiently be controlled within one experiment. The fit between subjective skills/abilities and adjusted task demands may trigger differences in neural activations compared to both control conditions that may support the experience of flow but do not necessarily represent the pure neuronal correlates of flow. Given the present paradigm with arithmetic tasks of different difficulty, some very recent advances in delineating different procedural steps in arithmetic problem solving may provide a fruitful approach to achieve further insight into this issue (e.g., Tschentscher and Hauk, 2016; van der Ven et al., 2016). For example, using temporally highly resolved magnetoencephalographic measures, Tschentscher and Hauk (2016) could demonstrate that different solution steps ranging from task encoding over rule and strategy selection to step-wise task execution were differently associated with desynchronizations in the alpha and beta band depending on task demands, and it may be these desynchronization differences that differently contribute to the experience of flow and correlated neural activations. Using hidden semi-Markov models multivariate pattern analysis of fMRI data, Anderson et al. (2016) recently revived a long standing discussion on serial multi stage process models as for example already suggested by Saul Sternberg in the 1960s (Sternberg, 1966). They could show that experimental manipulation of a mathematical problem solving task increased the duration of the planning stage as the method for solving the problem became less obvious. By contrast, duration for the solving stage increased with experimentally induced increase of calculations necessary to produce the final response. It is not unlikely that changes in stage durations may also add to the experience of flow and correlated neural activation in different brain regions.

However, as it presently stands these hypotheses cannot be tested with the actual task and study design. Furthermore, while the above ideas relate to task-related processual variations, contributions of other concomitant cognitive or affective processes may also remain undetected, and we feel unsure whether we will be ever in the position to get these contributions isolated, reliably and validly measured, and controlled. This, however, is not a shortcoming of the flow concept but has rather more to do with a still insufficient ontology and taxonomy of psychic functions and processes. As long as this framework is not readily existent, we therefore suggest to rely on a more operational definition of flow that can be derived from the optimum fit criterion. With the necessary fit between individual skills/abilities and task demands, the flow concept has a clear defining feature, and with both, decrease in self-referential processing and decrease in emotional arousal, it makes two clear and testable predictions including their neural correlates, i.e., the MPFC and the AMY with relative decreases in activation during flow that are significantly correlated with subjective experiences. In so far, the experience of flow during a task or action is not necessarily bound to any specific mental processing but may emerge with any task, given this optimum fit criterion is fulfilled.

## Author Contributions

MU conducted the experiment, analyzed the data, and wrote the manuscript. JK designed the study and wrote the manuscript. GG designed the study, analyzed the data, and wrote the manuscript.

## Conflict of Interest Statement

The authors declare that the research was conducted in the absence of any commercial or financial relationships that could be construed as a potential conflict of interest.
