# Supplementary material for: Dorsal Raphe Nucleus Down-Regulates Medial Prefrontal Cortex during Experience of Flow
Source: Front Behav Neurosci. 2016 Sep 5;10:169. doi: 10.3389/fnbeh.2016.00169 (PMC5011146; doi:10.3389/fnbeh.2016.00169)
Supplement: Supplementary file 1 [file Table_1.PDF]

## *Supplementary Material*

# Dorsal Raphe Nucleus Down-Regulates Medial Prefrontal Cortex during Experience of Flow

**Martin Ulrich*, Johannes Keller, Georg Grön**

*** Correspondence:** Martin Ulrich: martin.ulrich@uni-ulm.de

On the following pages the entire model space is presented, consisting of 64 Dynamic Causal Models (DCM), partitioned into eight families (indicated in parentheses). All models share identical endogenous connections (DCM.a) and direct input (DCM.c), but differ in modulatory effects (DCM.b) of experimental conditions "boredom", "flow", and "overload" on one or more (forward and/or backward) connections between the dorsal raphe nucleus (DRN) and the medial prefrontal cortex (MPFC), between the DRN and the amygdala, and between the MPFC and the amygdala. Presence/absence of a given connection/modulatory effect is coded by "1" and "0", respectively.

## Model 1 (member of family 1)

**DCM.a**

| from: / to: | Calcarine | DRN | MPFC | Amygdala |
|---|---|---|---|---|
| Calcarine | 1 | 1 | 1 | 1 |
| DRN | 1 | 1 | 1 | 1 |
| MPFC | 1 | 1 | 1 | 1 |
| Amygdala | 1 | 1 | 1 | 1 |

**DCM.b(:,:,1)**
***[Boredom]***

| from: / to: | Calcarine | DRN | MPFC | Amygdala |
|---|---|---|---|---|
| Calcarine | 0 | 0 | 0 | 0 |
| DRN | 0 | 0 | 1 | 1 |
| MPFC | 0 | 1 | 0 | 1 |
| Amygdala | 0 | 1 | 1 | 0 |

**DCM.b(:,:,2)**
***[Flow]***

| from: / to: | Calcarine | DRN | MPFC | Amygdala |
|---|---|---|---|---|
| Calcarine | 0 | 0 | 0 | 0 |
| DRN | 0 | 0 | 1 | 1 |
| MPFC | 0 | 1 | 0 | 1 |
| Amygdala | 0 | 1 | 1 | 0 |

**DCM.b(:,:,3)**
***[Overload]***

| from: / to: | Calcarine | DRN | MPFC | Amygdala |
|---|---|---|---|---|
| Calcarine | 0 | 0 | 0 | 0 |
| DRN | 0 | 0 | 1 | 1 |
| MPFC | 0 | 1 | 0 | 1 |
| Amygdala | 0 | 1 | 1 | 0 |

**DCM.c**

| | Boredom | Flow | Overload |
|---|---|---|---|
| Calcarine | 1 | 1 | 1 |
| DRN | 0 | 0 | 0 |
| MPFC | 0 | 0 | 0 |
| Amygdala | 0 | 0 | 0 |

# Model 2  (member of family 1)

**DCM.a**

| *from:* / *to:* | Calcarine | DRN | MPFC | Amygdala |
|---|---|---|---|---|
| Calcarine | 1 | 1 | 1 | 1 |
| DRN | 1 | 1 | 1 | 1 |
| MPFC | 1 | 1 | 1 | 1 |
| Amygdala | 1 | 1 | 1 | 1 |

**DCM.b(:,:,1)**
***[Boredom]***

| *from:* / *to:* | Calcarine | DRN | MPFC | Amygdala |
|---|---|---|---|---|
| Calcarine | 0 | 0 | 0 | 0 |
| DRN | 0 | 0 | 1 | 1 |
| MPFC | 0 | 1 | 0 | 0 |
| Amygdala | 0 | 1 | 1 | 0 |

**DCM.b(:,:,2)**
***[Flow]***

| *from:* / *to:* | Calcarine | DRN | MPFC | Amygdala |
|---|---|---|---|---|
| Calcarine | 0 | 0 | 0 | 0 |
| DRN | 0 | 0 | 1 | 1 |
| MPFC | 0 | 1 | 0 | 0 |
| Amygdala | 0 | 1 | 1 | 0 |

**DCM.b(:,:,3)**
***[Overload]***

| *from:* / *to:* | Calcarine | DRN | MPFC | Amygdala |
|---|---|---|---|---|
| Calcarine | 0 | 0 | 0 | 0 |
| DRN | 0 | 0 | 1 | 1 |
| MPFC | 0 | 1 | 0 | 0 |
| Amygdala | 0 | 1 | 1 | 0 |

**DCM.c**

| | Boredom | Flow | Overload |
|---|---|---|---|
| Calcarine | 1 | 1 | 1 |
| DRN | 0 | 0 | 0 |
| MPFC | 0 | 0 | 0 |
| Amygdala | 0 | 0 | 0 |

## Model 3 (member of family 1)

**DCM.a**

| *from:* / *to:* | Calcarine | DRN | MPFC | Amygdala |
|---|---|---|---|---|
| Calcarine | 1 | 1 | 1 | 1 |
| DRN | 1 | 1 | 1 | 1 |
| MPFC | 1 | 1 | 1 | 1 |
| Amygdala | 1 | 1 | 1 | 1 |

**DCM.b(:,:,1)**
***[Boredom]***

| *from:* / *to:* | Calcarine | DRN | MPFC | Amygdala |
|---|---|---|---|---|
| Calcarine | 0 | 0 | 0 | 0 |
| DRN | 0 | 0 | 1 | 0 |
| MPFC | 0 | 1 | 0 | 1 |
| Amygdala | 0 | 1 | 1 | 0 |

**DCM.b(:,:,2)**
***[Flow]***

| *from:* / *to:* | Calcarine | DRN | MPFC | Amygdala |
|---|---|---|---|---|
| Calcarine | 0 | 0 | 0 | 0 |
| DRN | 0 | 0 | 1 | 0 |
| MPFC | 0 | 1 | 0 | 1 |
| Amygdala | 0 | 1 | 1 | 0 |

**DCM.b(:,:,3)**
***[Overload]***

| *from:* / *to:* | Calcarine | DRN | MPFC | Amygdala |
|---|---|---|---|---|
| Calcarine | 0 | 0 | 0 | 0 |
| DRN | 0 | 0 | 1 | 0 |
| MPFC | 0 | 1 | 0 | 1 |
| Amygdala | 0 | 1 | 1 | 0 |

**DCM.c**

| | Boredom | Flow | Overload |
|---|---|---|---|
| Calcarine | 1 | 1 | 1 |
| DRN | 0 | 0 | 0 |
| MPFC | 0 | 0 | 0 |
| Amygdala | 0 | 0 | 0 |

## Model 4 (member of family 1)

**DCM.a**

| from: / to: | Calcarine | DRN | MPFC | Amygdala |
|---|---|---|---|---|
| Calcarine | 1 | 1 | 1 | 1 |
| DRN | 1 | 1 | 1 | 1 |
| MPFC | 1 | 1 | 1 | 1 |
| Amygdala | 1 | 1 | 1 | 1 |

**DCM.b(:,:,1)**
***[Boredom]***

| from: / to: | Calcarine | DRN | MPFC | Amygdala |
|---|---|---|---|---|
| Calcarine | 0 | 0 | 0 | 0 |
| DRN | 0 | 0 | 1 | 0 |
| MPFC | 0 | 1 | 0 | 0 |
| Amygdala | 0 | 1 | 1 | 0 |

**DCM.b(:,:,2)**
***[Flow]***

| from: / to: | Calcarine | DRN | MPFC | Amygdala |
|---|---|---|---|---|
| Calcarine | 0 | 0 | 0 | 0 |
| DRN | 0 | 0 | 1 | 0 |
| MPFC | 0 | 1 | 0 | 0 |
| Amygdala | 0 | 1 | 1 | 0 |

**DCM.b(:,:,3)**
***[Overload]***

| from: / to: | Calcarine | DRN | MPFC | Amygdala |
|---|---|---|---|---|
| Calcarine | 0 | 0 | 0 | 0 |
| DRN | 0 | 0 | 1 | 0 |
| MPFC | 0 | 1 | 0 | 0 |
| Amygdala | 0 | 1 | 1 | 0 |

**DCM.c**

| | Boredom | Flow | Overload |
|---|---|---|---|
| Calcarine | 1 | 1 | 1 |
| DRN | 0 | 0 | 0 |
| MPFC | 0 | 0 | 0 |
| Amygdala | 0 | 0 | 0 |

## Model 5 (member of family 1)

**DCM.a**

| from: / to: | Calcarine | DRN | MPFC | Amygdala |
|---|---|---|---|---|
| Calcarine | 1 | 1 | 1 | 1 |
| DRN | 1 | 1 | 1 | 1 |
| MPFC | 1 | 1 | 1 | 1 |
| Amygdala | 1 | 1 | 1 | 1 |

**DCM.b(:,:,1)**
***[Boredom]***

| from: / to: | Calcarine | DRN | MPFC | Amygdala |
|---|---|---|---|---|
| Calcarine | 0 | 0 | 0 | 0 |
| DRN | 0 | 0 | 1 | 1 |
| MPFC | 0 | 1 | 0 | 1 |
| Amygdala | 0 | 1 | 0 | 0 |

**DCM.b(:,:,2)**
***[Flow]***

| from: / to: | Calcarine | DRN | MPFC | Amygdala |
|---|---|---|---|---|
| Calcarine | 0 | 0 | 0 | 0 |
| DRN | 0 | 0 | 1 | 1 |
| MPFC | 0 | 1 | 0 | 1 |
| Amygdala | 0 | 1 | 0 | 0 |

**DCM.b(:,:,3)**
***[Overload]***

| from: / to: | Calcarine | DRN | MPFC | Amygdala |
|---|---|---|---|---|
| Calcarine | 0 | 0 | 0 | 0 |
| DRN | 0 | 0 | 1 | 1 |
| MPFC | 0 | 1 | 0 | 1 |
| Amygdala | 0 | 1 | 0 | 0 |

**DCM.c**

| | Boredom | Flow | Overload |
|---|---|---|---|
| Calcarine | 1 | 1 | 1 |
| DRN | 0 | 0 | 0 |
| MPFC | 0 | 0 | 0 |
| Amygdala | 0 | 0 | 0 |

## Model 6 (member of family 1)

**DCM.a**

| *from:* / *to:* | Calcarine | DRN | MPFC | Amygdala |
|---|---|---|---|---|
| Calcarine | 1 | 1 | 1 | 1 |
| DRN | 1 | 1 | 1 | 1 |
| MPFC | 1 | 1 | 1 | 1 |
| Amygdala | 1 | 1 | 1 | 1 |

**DCM.b(:,:,1)**
***[Boredom]***

| *from:* / *to:* | Calcarine | DRN | MPFC | Amygdala |
|---|---|---|---|---|
| Calcarine | 0 | 0 | 0 | 0 |
| DRN | 0 | 0 | 1 | 0 |
| MPFC | 0 | 1 | 0 | 1 |
| Amygdala | 0 | 1 | 0 | 0 |

**DCM.b(:,:,2)**
***[Flow]***

| *from:* / *to:* | Calcarine | DRN | MPFC | Amygdala |
|---|---|---|---|---|
| Calcarine | 0 | 0 | 0 | 0 |
| DRN | 0 | 0 | 1 | 0 |
| MPFC | 0 | 1 | 0 | 1 |
| Amygdala | 0 | 1 | 0 | 0 |

**DCM.b(:,:,3)**
***[Overload]***

| *from:* / *to:* | Calcarine | DRN | MPFC | Amygdala |
|---|---|---|---|---|
| Calcarine | 0 | 0 | 0 | 0 |
| DRN | 0 | 0 | 1 | 0 |
| MPFC | 0 | 1 | 0 | 1 |
| Amygdala | 0 | 1 | 0 | 0 |

**DCM.c**

| | Boredom | Flow | Overload |
|---|---|---|---|
| Calcarine | 1 | 1 | 1 |
| DRN | 0 | 0 | 0 |
| MPFC | 0 | 0 | 0 |
| Amygdala | 0 | 0 | 0 |

# Model 7  (member of family 1)

**DCM.a**

| *from:* / *to:* | Calcarine | DRN | MPFC | Amygdala |
|---|---|---|---|---|
| Calcarine | 1 | 1 | 1 | 1 |
| DRN | 1 | 1 | 1 | 1 |
| MPFC | 1 | 1 | 1 | 1 |
| Amygdala | 1 | 1 | 1 | 1 |

**DCM.b(:,:,1)**
***[Boredom]***

| *from:* / *to:* | Calcarine | DRN | MPFC | Amygdala |
|---|---|---|---|---|
| Calcarine | 0 | 0 | 0 | 0 |
| DRN | 0 | 0 | 0 | 1 |
| MPFC | 0 | 1 | 0 | 1 |
| Amygdala | 0 | 1 | 1 | 0 |

**DCM.b(:,:,2)**
***[Flow]***

| *from:* / *to:* | Calcarine | DRN | MPFC | Amygdala |
|---|---|---|---|---|
| Calcarine | 0 | 0 | 0 | 0 |
| DRN | 0 | 0 | 0 | 1 |
| MPFC | 0 | 1 | 0 | 1 |
| Amygdala | 0 | 1 | 1 | 0 |

**DCM.b(:,:,3)**
***[Overload]***

| *from:* / *to:* | Calcarine | DRN | MPFC | Amygdala |
|---|---|---|---|---|
| Calcarine | 0 | 0 | 0 | 0 |
| DRN | 0 | 0 | 0 | 1 |
| MPFC | 0 | 1 | 0 | 1 |
| Amygdala | 0 | 1 | 1 | 0 |

**DCM.c**

| | Boredom | Flow | Overload |
|---|---|---|---|
| Calcarine | 1 | 1 | 1 |
| DRN | 0 | 0 | 0 |
| MPFC | 0 | 0 | 0 |
| Amygdala | 0 | 0 | 0 |

## Model 8 (member of family 1)

**DCM.a**

| *from:* / *to:* | Calcarine | DRN | MPFC | Amygdala |
|---|---|---|---|---|
| Calcarine | 1 | 1 | 1 | 1 |
| DRN | 1 | 1 | 1 | 1 |
| MPFC | 1 | 1 | 1 | 1 |
| Amygdala | 1 | 1 | 1 | 1 |

**DCM.b(:,:,1)**
***[Boredom]***

| *from:* / *to:* | Calcarine | DRN | MPFC | Amygdala |
|---|---|---|---|---|
| Calcarine | 0 | 0 | 0 | 0 |
| DRN | 0 | 0 | 0 | 1 |
| MPFC | 0 | 1 | 0 | 0 |
| Amygdala | 0 | 1 | 1 | 0 |

**DCM.b(:,:,2)**
***[Flow]***

| *from:* / *to:* | Calcarine | DRN | MPFC | Amygdala |
|---|---|---|---|---|
| Calcarine | 0 | 0 | 0 | 0 |
| DRN | 0 | 0 | 0 | 1 |
| MPFC | 0 | 1 | 0 | 0 |
| Amygdala | 0 | 1 | 1 | 0 |

**DCM.b(:,:,3)**
***[Overload]***

| *from:* / *to:* | Calcarine | DRN | MPFC | Amygdala |
|---|---|---|---|---|
| Calcarine | 0 | 0 | 0 | 0 |
| DRN | 0 | 0 | 0 | 1 |
| MPFC | 0 | 1 | 0 | 0 |
| Amygdala | 0 | 1 | 1 | 0 |

**DCM.c**

| | Boredom | Flow | Overload |
|---|---|---|---|
| Calcarine | 1 | 1 | 1 |
| DRN | 0 | 0 | 0 |
| MPFC | 0 | 0 | 0 |
| Amygdala | 0 | 0 | 0 |

## Model 9  (member of family 1)

**DCM.a**

| *to:* \ *from:* | Calcarine | DRN | MPFC | Amygdala |
|---|---|---|---|---|
| Calcarine | 1 | 1 | 1 | 1 |
| DRN | 1 | 1 | 1 | 1 |
| MPFC | 1 | 1 | 1 | 1 |
| Amygdala | 1 | 1 | 1 | 1 |

**DCM.b(:,:,1)**
***[Boredom]***

| *to:* \ *from:* | Calcarine | DRN | MPFC | Amygdala |
|---|---|---|---|---|
| Calcarine | 0 | 0 | 0 | 0 |
| DRN | 0 | 0 | 0 | 0 |
| MPFC | 0 | 1 | 0 | 1 |
| Amygdala | 0 | 1 | 1 | 0 |

**DCM.b(:,:,2)**
***[Flow]***

| *to:* \ *from:* | Calcarine | DRN | MPFC | Amygdala |
|---|---|---|---|---|
| Calcarine | 0 | 0 | 0 | 0 |
| DRN | 0 | 0 | 0 | 0 |
| MPFC | 0 | 1 | 0 | 1 |
| Amygdala | 0 | 1 | 1 | 0 |

**DCM.b(:,:,3)**
***[Overload]***

| *to:* \ *from:* | Calcarine | DRN | MPFC | Amygdala |
|---|---|---|---|---|
| Calcarine | 0 | 0 | 0 | 0 |
| DRN | 0 | 0 | 0 | 0 |
| MPFC | 0 | 1 | 0 | 1 |
| Amygdala | 0 | 1 | 1 | 0 |

**DCM.c**

| | Boredom | Flow | Overload |
|---|---|---|---|
| Calcarine | 1 | 1 | 1 |
| DRN | 0 | 0 | 0 |
| MPFC | 0 | 0 | 0 |
| Amygdala | 0 | 0 | 0 |

## Model 10 (member of family 1)

**DCM.a**

| from: / to: | Calcarine | DRN | MPFC | Amygdala |
|---|---|---|---|---|
| Calcarine | 1 | 1 | 1 | 1 |
| DRN | 1 | 1 | 1 | 1 |
| MPFC | 1 | 1 | 1 | 1 |
| Amygdala | 1 | 1 | 1 | 1 |

**DCM.b(:,:,1)**
***[Boredom]***

| from: / to: | Calcarine | DRN | MPFC | Amygdala |
|---|---|---|---|---|
| Calcarine | 0 | 0 | 0 | 0 |
| DRN | 0 | 0 | 0 | 0 |
| MPFC | 0 | 1 | 0 | 0 |
| Amygdala | 0 | 1 | 1 | 0 |

**DCM.b(:,:,2)**
***[Flow]***

| from: / to: | Calcarine | DRN | MPFC | Amygdala |
|---|---|---|---|---|
| Calcarine | 0 | 0 | 0 | 0 |
| DRN | 0 | 0 | 0 | 0 |
| MPFC | 0 | 1 | 0 | 0 |
| Amygdala | 0 | 1 | 1 | 0 |

**DCM.b(:,:,3)**
***[Overload]***

| from: / to: | Calcarine | DRN | MPFC | Amygdala |
|---|---|---|---|---|
| Calcarine | 0 | 0 | 0 | 0 |
| DRN | 0 | 0 | 0 | 0 |
| MPFC | 0 | 1 | 0 | 0 |
| Amygdala | 0 | 1 | 1 | 0 |

**DCM.c**

| | Boredom | Flow | Overload |
|---|---|---|---|
| Calcarine | 1 | 1 | 1 |
| DRN | 0 | 0 | 0 |
| MPFC | 0 | 0 | 0 |
| Amygdala | 0 | 0 | 0 |

## Model 11 (member of family 1)

**DCM.a**

| *from:* / *to:* | Calcarine | DRN | MPFC | Amygdala |
|---|---|---|---|---|
| Calcarine | 1 | 1 | 1 | 1 |
| DRN | 1 | 1 | 1 | 1 |
| MPFC | 1 | 1 | 1 | 1 |
| Amygdala | 1 | 1 | 1 | 1 |

**DCM.b(:,:,1)**
***[Boredom]***

| *from:* / *to:* | Calcarine | DRN | MPFC | Amygdala |
|---|---|---|---|---|
| Calcarine | 0 | 0 | 0 | 0 |
| DRN | 0 | 0 | 0 | 1 |
| MPFC | 0 | 1 | 0 | 1 |
| Amygdala | 0 | 1 | 0 | 0 |

**DCM.b(:,:,2)**
***[Flow]***

| *from:* / *to:* | Calcarine | DRN | MPFC | Amygdala |
|---|---|---|---|---|
| Calcarine | 0 | 0 | 0 | 0 |
| DRN | 0 | 0 | 0 | 1 |
| MPFC | 0 | 1 | 0 | 1 |
| Amygdala | 0 | 1 | 0 | 0 |

**DCM.b(:,:,3)**
***[Overload]***

| *from:* / *to:* | Calcarine | DRN | MPFC | Amygdala |
|---|---|---|---|---|
| Calcarine | 0 | 0 | 0 | 0 |
| DRN | 0 | 0 | 0 | 1 |
| MPFC | 0 | 1 | 0 | 1 |
| Amygdala | 0 | 1 | 0 | 0 |

**DCM.c**

| | Boredom | Flow | Overload |
|---|---|---|---|
| Calcarine | 1 | 1 | 1 |
| DRN | 0 | 0 | 0 |
| MPFC | 0 | 0 | 0 |
| Amygdala | 0 | 0 | 0 |

# Model 12 (member of family 1)

**DCM.a**

| *from:* / *to:* | Calcarine | DRN | MPFC | Amygdala |
|---|---|---|---|---|
| Calcarine | 1 | 1 | 1 | 1 |
| DRN | 1 | 1 | 1 | 1 |
| MPFC | 1 | 1 | 1 | 1 |
| Amygdala | 1 | 1 | 1 | 1 |

**DCM.b(:,:,1)**
***[Boredom]***

| *from:* / *to:* | Calcarine | DRN | MPFC | Amygdala |
|---|---|---|---|---|
| Calcarine | 0 | 0 | 0 | 0 |
| DRN | 0 | 0 | 0 | 0 |
| MPFC | 0 | 1 | 0 | 1 |
| Amygdala | 0 | 1 | 0 | 0 |

**DCM.b(:,:,2)**
***[Flow]***

| *from:* / *to:* | Calcarine | DRN | MPFC | Amygdala |
|---|---|---|---|---|
| Calcarine | 0 | 0 | 0 | 0 |
| DRN | 0 | 0 | 0 | 0 |
| MPFC | 0 | 1 | 0 | 1 |
| Amygdala | 0 | 1 | 0 | 0 |

**DCM.b(:,:,3)**
***[Overload]***

| *from:* / *to:* | Calcarine | DRN | MPFC | Amygdala |
|---|---|---|---|---|
| Calcarine | 0 | 0 | 0 | 0 |
| DRN | 0 | 0 | 0 | 0 |
| MPFC | 0 | 1 | 0 | 1 |
| Amygdala | 0 | 1 | 0 | 0 |

**DCM.c**

| | Boredom | Flow | Overload |
|---|---|---|---|
| Calcarine | 1 | 1 | 1 |
| DRN | 0 | 0 | 0 |
| MPFC | 0 | 0 | 0 |
| Amygdala | 0 | 0 | 0 |

## Model 13 (member of family 1)

**DCM.a**

| *from:* / *to:* | Calcarine | DRN | MPFC | Amygdala |
|---|---|---|---|---|
| Calcarine | 1 | 1 | 1 | 1 |
| DRN | 1 | 1 | 1 | 1 |
| MPFC | 1 | 1 | 1 | 1 |
| Amygdala | 1 | 1 | 1 | 1 |

**DCM.b(:,:,1)**
***[Boredom]***

| *from:* / *to:* | Calcarine | DRN | MPFC | Amygdala |
|---|---|---|---|---|
| Calcarine | 0 | 0 | 0 | 0 |
| DRN | 0 | 0 | 1 | 1 |
| MPFC | 0 | 1 | 0 | 1 |
| Amygdala | 0 | 0 | 1 | 0 |

**DCM.b(:,:,2)**
***[Flow]***

| *from:* / *to:* | Calcarine | DRN | MPFC | Amygdala |
|---|---|---|---|---|
| Calcarine | 0 | 0 | 0 | 0 |
| DRN | 0 | 0 | 1 | 1 |
| MPFC | 0 | 1 | 0 | 1 |
| Amygdala | 0 | 0 | 1 | 0 |

**DCM.b(:,:,3)**
***[Overload]***

| *from:* / *to:* | Calcarine | DRN | MPFC | Amygdala |
|---|---|---|---|---|
| Calcarine | 0 | 0 | 0 | 0 |
| DRN | 0 | 0 | 1 | 1 |
| MPFC | 0 | 1 | 0 | 1 |
| Amygdala | 0 | 0 | 1 | 0 |

**DCM.c**

| | Boredom | Flow | Overload |
|---|---|---|---|
| Calcarine | 1 | 1 | 1 |
| DRN | 0 | 0 | 0 |
| MPFC | 0 | 0 | 0 |
| Amygdala | 0 | 0 | 0 |

## Model 14  (member of family 1)

**DCM.a**

| *from:* / *to:* | Calcarine | DRN | MPFC | Amygdala |
|---|---|---|---|---|
| Calcarine | 1 | 1 | 1 | 1 |
| DRN | 1 | 1 | 1 | 1 |
| MPFC | 1 | 1 | 1 | 1 |
| Amygdala | 1 | 1 | 1 | 1 |

**DCM.b(:,:,1)**
***[Boredom]***

| *from:* / *to:* | Calcarine | DRN | MPFC | Amygdala |
|---|---|---|---|---|
| Calcarine | 0 | 0 | 0 | 0 |
| DRN | 0 | 0 | 1 | 1 |
| MPFC | 0 | 1 | 0 | 0 |
| Amygdala | 0 | 0 | 1 | 0 |

**DCM.b(:,:,2)**
***[Flow]***

| *from:* / *to:* | Calcarine | DRN | MPFC | Amygdala |
|---|---|---|---|---|
| Calcarine | 0 | 0 | 0 | 0 |
| DRN | 0 | 0 | 1 | 1 |
| MPFC | 0 | 1 | 0 | 0 |
| Amygdala | 0 | 0 | 1 | 0 |

**DCM.b(:,:,3)**
***[Overload]***

| *from:* / *to:* | Calcarine | DRN | MPFC | Amygdala |
|---|---|---|---|---|
| Calcarine | 0 | 0 | 0 | 0 |
| DRN | 0 | 0 | 1 | 1 |
| MPFC | 0 | 1 | 0 | 0 |
| Amygdala | 0 | 0 | 1 | 0 |

**DCM.c**

| | Boredom | Flow | Overload |
|---|---|---|---|
| Calcarine | 1 | 1 | 1 |
| DRN | 0 | 0 | 0 |
| MPFC | 0 | 0 | 0 |
| Amygdala | 0 | 0 | 0 |

## Model 15 (member of family 1)

**DCM.a**

| *from:* / *to:* | Calcarine | DRN | MPFC | Amygdala |
|---|---|---|---|---|
| Calcarine | 1 | 1 | 1 | 1 |
| DRN | 1 | 1 | 1 | 1 |
| MPFC | 1 | 1 | 1 | 1 |
| Amygdala | 1 | 1 | 1 | 1 |

**DCM.b(:,:,1)**
***[Boredom]***

| *from:* / *to:* | Calcarine | DRN | MPFC | Amygdala |
|---|---|---|---|---|
| Calcarine | 0 | 0 | 0 | 0 |
| DRN | 0 | 0 | 1 | 1 |
| MPFC | 0 | 1 | 0 | 1 |
| Amygdala | 0 | 0 | 0 | 0 |

**DCM.b(:,:,2)**
***[Flow]***

| *from:* / *to:* | Calcarine | DRN | MPFC | Amygdala |
|---|---|---|---|---|
| Calcarine | 0 | 0 | 0 | 0 |
| DRN | 0 | 0 | 1 | 1 |
| MPFC | 0 | 1 | 0 | 1 |
| Amygdala | 0 | 0 | 0 | 0 |

**DCM.b(:,:,3)**
***[Overload]***

| *from:* / *to:* | Calcarine | DRN | MPFC | Amygdala |
|---|---|---|---|---|
| Calcarine | 0 | 0 | 0 | 0 |
| DRN | 0 | 0 | 1 | 1 |
| MPFC | 0 | 1 | 0 | 1 |
| Amygdala | 0 | 0 | 0 | 0 |

**DCM.c**

| | Boredom | Flow | Overload |
|---|---|---|---|
| Calcarine | 1 | 1 | 1 |
| DRN | 0 | 0 | 0 |
| MPFC | 0 | 0 | 0 |
| Amygdala | 0 | 0 | 0 |

## Model 16 (member of family 1)

**DCM.a**

| *to:* \ *from:* | Calcarine | DRN | MPFC | Amygdala |
|---|---|---|---|---|
| Calcarine | 1 | 1 | 1 | 1 |
| DRN | 1 | 1 | 1 | 1 |
| MPFC | 1 | 1 | 1 | 1 |
| Amygdala | 1 | 1 | 1 | 1 |

**DCM.b(:,:,1)**
***[Boredom]***

| *to:* \ *from:* | Calcarine | DRN | MPFC | Amygdala |
|---|---|---|---|---|
| Calcarine | 0 | 0 | 0 | 0 |
| DRN | 0 | 0 | 0 | 1 |
| MPFC | 0 | 1 | 0 | 1 |
| Amygdala | 0 | 0 | 1 | 0 |

**DCM.b(:,:,2)**
***[Flow]***

| *to:* \ *from:* | Calcarine | DRN | MPFC | Amygdala |
|---|---|---|---|---|
| Calcarine | 0 | 0 | 0 | 0 |
| DRN | 0 | 0 | 0 | 1 |
| MPFC | 0 | 1 | 0 | 1 |
| Amygdala | 0 | 0 | 1 | 0 |

**DCM.b(:,:,3)**
***[Overload]***

| *to:* \ *from:* | Calcarine | DRN | MPFC | Amygdala |
|---|---|---|---|---|
| Calcarine | 0 | 0 | 0 | 0 |
| DRN | 0 | 0 | 0 | 1 |
| MPFC | 0 | 1 | 0 | 1 |
| Amygdala | 0 | 0 | 1 | 0 |

**DCM.c**

| | Boredom | Flow | Overload |
|---|---|---|---|
| Calcarine | 1 | 1 | 1 |
| DRN | 0 | 0 | 0 |
| MPFC | 0 | 0 | 0 |
| Amygdala | 0 | 0 | 0 |

## Model 17 (member of family 1)

**DCM.a**

| from: / to: | Calcarine | DRN | MPFC | Amygdala |
|---|---|---|---|---|
| Calcarine | 1 | 1 | 1 | 1 |
| DRN | 1 | 1 | 1 | 1 |
| MPFC | 1 | 1 | 1 | 1 |
| Amygdala | 1 | 1 | 1 | 1 |

**DCM.b(:,:,1)**
***[Boredom]***

| from: / to: | Calcarine | DRN | MPFC | Amygdala |
|---|---|---|---|---|
| Calcarine | 0 | 0 | 0 | 0 |
| DRN | 0 | 0 | 0 | 1 |
| MPFC | 0 | 1 | 0 | 0 |
| Amygdala | 0 | 0 | 1 | 0 |

**DCM.b(:,:,2)**
***[Flow]***

| from: / to: | Calcarine | DRN | MPFC | Amygdala |
|---|---|---|---|---|
| Calcarine | 0 | 0 | 0 | 0 |
| DRN | 0 | 0 | 0 | 1 |
| MPFC | 0 | 1 | 0 | 0 |
| Amygdala | 0 | 0 | 1 | 0 |

**DCM.b(:,:,3)**
***[Overload]***

| from: / to: | Calcarine | DRN | MPFC | Amygdala |
|---|---|---|---|---|
| Calcarine | 0 | 0 | 0 | 0 |
| DRN | 0 | 0 | 0 | 1 |
| MPFC | 0 | 1 | 0 | 0 |
| Amygdala | 0 | 0 | 1 | 0 |

**DCM.c**

| | Boredom | Flow | Overload |
|---|---|---|---|
| Calcarine | 1 | 1 | 1 |
| DRN | 0 | 0 | 0 |
| MPFC | 0 | 0 | 0 |
| Amygdala | 0 | 0 | 0 |

## Model 18 (member of family 1)

**DCM.a**

| *from:* / *to:* | Calcarine | DRN | MPFC | Amygdala |
|---|---|---|---|---|
| Calcarine | 1 | 1 | 1 | 1 |
| DRN | 1 | 1 | 1 | 1 |
| MPFC | 1 | 1 | 1 | 1 |
| Amygdala | 1 | 1 | 1 | 1 |

**DCM.b(:,:,1)**
***[Boredom]***

| *from:* / *to:* | Calcarine | DRN | MPFC | Amygdala |
|---|---|---|---|---|
| Calcarine | 0 | 0 | 0 | 0 |
| DRN | 0 | 0 | 0 | 1 |
| MPFC | 0 | 1 | 0 | 1 |
| Amygdala | 0 | 0 | 0 | 0 |

**DCM.b(:,:,2)**
***[Flow]***

| *from:* / *to:* | Calcarine | DRN | MPFC | Amygdala |
|---|---|---|---|---|
| Calcarine | 0 | 0 | 0 | 0 |
| DRN | 0 | 0 | 0 | 1 |
| MPFC | 0 | 1 | 0 | 1 |
| Amygdala | 0 | 0 | 0 | 0 |

**DCM.b(:,:,3)**
***[Overload]***

| *from:* / *to:* | Calcarine | DRN | MPFC | Amygdala |
|---|---|---|---|---|
| Calcarine | 0 | 0 | 0 | 0 |
| DRN | 0 | 0 | 0 | 1 |
| MPFC | 0 | 1 | 0 | 1 |
| Amygdala | 0 | 0 | 0 | 0 |

**DCM.c**

| | Boredom | Flow | Overload |
|---|---|---|---|
| Calcarine | 1 | 1 | 1 |
| DRN | 0 | 0 | 0 |
| MPFC | 0 | 0 | 0 |
| Amygdala | 0 | 0 | 0 |

## Model 19 (member of family 1)

**DCM.a**

| *from:* / *to:* | Calcarine | DRN | MPFC | Amygdala |
|---|---|---|---|---|
| Calcarine | 1 | 1 | 1 | 1 |
| DRN | 1 | 1 | 1 | 1 |
| MPFC | 1 | 1 | 1 | 1 |
| Amygdala | 1 | 1 | 1 | 1 |

**DCM.b(:,:,1)**
***[Boredom]***

| *from:* / *to:* | Calcarine | DRN | MPFC | Amygdala |
|---|---|---|---|---|
| Calcarine | 0 | 0 | 0 | 0 |
| DRN | 0 | 0 | 1 | 1 |
| MPFC | 0 | 0 | 0 | 1 |
| Amygdala | 0 | 1 | 1 | 0 |

**DCM.b(:,:,2)**
***[Flow]***

| *from:* / *to:* | Calcarine | DRN | MPFC | Amygdala |
|---|---|---|---|---|
| Calcarine | 0 | 0 | 0 | 0 |
| DRN | 0 | 0 | 1 | 1 |
| MPFC | 0 | 0 | 0 | 1 |
| Amygdala | 0 | 1 | 1 | 0 |

**DCM.b(:,:,3)**
***[Overload]***

| *from:* / *to:* | Calcarine | DRN | MPFC | Amygdala |
|---|---|---|---|---|
| Calcarine | 0 | 0 | 0 | 0 |
| DRN | 0 | 0 | 1 | 1 |
| MPFC | 0 | 0 | 0 | 1 |
| Amygdala | 0 | 1 | 1 | 0 |

**DCM.c**

| | Boredom | Flow | Overload |
|---|---|---|---|
| Calcarine | 1 | 1 | 1 |
| DRN | 0 | 0 | 0 |
| MPFC | 0 | 0 | 0 |
| Amygdala | 0 | 0 | 0 |

## Model 20 (member of family 1)

**DCM.a**

| to: \ from: | Calcarine | DRN | MPFC | Amygdala |
|---|---|---|---|---|
| Calcarine | 1 | 1 | 1 | 1 |
| DRN | 1 | 1 | 1 | 1 |
| MPFC | 1 | 1 | 1 | 1 |
| Amygdala | 1 | 1 | 1 | 1 |

**DCM.b(:,:,1)**
***[Boredom]***

| to: \ from: | Calcarine | DRN | MPFC | Amygdala |
|---|---|---|---|---|
| Calcarine | 0 | 0 | 0 | 0 |
| DRN | 0 | 0 | 1 | 1 |
| MPFC | 0 | 0 | 0 | 0 |
| Amygdala | 0 | 1 | 1 | 0 |

**DCM.b(:,:,2)**
***[Flow]***

| to: \ from: | Calcarine | DRN | MPFC | Amygdala |
|---|---|---|---|---|
| Calcarine | 0 | 0 | 0 | 0 |
| DRN | 0 | 0 | 1 | 1 |
| MPFC | 0 | 0 | 0 | 0 |
| Amygdala | 0 | 1 | 1 | 0 |

**DCM.b(:,:,3)**
***[Overload]***

| to: \ from: | Calcarine | DRN | MPFC | Amygdala |
|---|---|---|---|---|
| Calcarine | 0 | 0 | 0 | 0 |
| DRN | 0 | 0 | 1 | 1 |
| MPFC | 0 | 0 | 0 | 0 |
| Amygdala | 0 | 1 | 1 | 0 |

**DCM.c**

| | Boredom | Flow | Overload |
|---|---|---|---|
| Calcarine | 1 | 1 | 1 |
| DRN | 0 | 0 | 0 |
| MPFC | 0 | 0 | 0 |
| Amygdala | 0 | 0 | 0 |

## Model 21 (member of family 1)

**DCM.a**

| *from:* / *to:* | Calcarine | DRN | MPFC | Amygdala |
|---|---|---|---|---|
| Calcarine | 1 | 1 | 1 | 1 |
| DRN | 1 | 1 | 1 | 1 |
| MPFC | 1 | 1 | 1 | 1 |
| Amygdala | 1 | 1 | 1 | 1 |

**DCM.b(:,:,1)**
***[Boredom]***

| *from:* / *to:* | Calcarine | DRN | MPFC | Amygdala |
|---|---|---|---|---|
| Calcarine | 0 | 0 | 0 | 0 |
| DRN | 0 | 0 | 1 | 0 |
| MPFC | 0 | 0 | 0 | 1 |
| Amygdala | 0 | 1 | 1 | 0 |

**DCM.b(:,:,2)**
***[Flow]***

| *from:* / *to:* | Calcarine | DRN | MPFC | Amygdala |
|---|---|---|---|---|
| Calcarine | 0 | 0 | 0 | 0 |
| DRN | 0 | 0 | 1 | 0 |
| MPFC | 0 | 0 | 0 | 1 |
| Amygdala | 0 | 1 | 1 | 0 |

**DCM.b(:,:,3)**
***[Overload]***

| *from:* / *to:* | Calcarine | DRN | MPFC | Amygdala |
|---|---|---|---|---|
| Calcarine | 0 | 0 | 0 | 0 |
| DRN | 0 | 0 | 1 | 0 |
| MPFC | 0 | 0 | 0 | 1 |
| Amygdala | 0 | 1 | 1 | 0 |

**DCM.c**

| | Boredom | Flow | Overload |
|---|---|---|---|
| Calcarine | 1 | 1 | 1 |
| DRN | 0 | 0 | 0 |
| MPFC | 0 | 0 | 0 |
| Amygdala | 0 | 0 | 0 |

## Model 22 (member of family 1)

**DCM.a**

| *from:* / *to:* | Calcarine | DRN | MPFC | Amygdala |
|---|---|---|---|---|
| Calcarine | 1 | 1 | 1 | 1 |
| DRN | 1 | 1 | 1 | 1 |
| MPFC | 1 | 1 | 1 | 1 |
| Amygdala | 1 | 1 | 1 | 1 |

**DCM.b(:,:,1)**
***[Boredom]***

| *from:* / *to:* | Calcarine | DRN | MPFC | Amygdala |
|---|---|---|---|---|
| Calcarine | 0 | 0 | 0 | 0 |
| DRN | 0 | 0 | 1 | 0 |
| MPFC | 0 | 0 | 0 | 0 |
| Amygdala | 0 | 1 | 1 | 0 |

**DCM.b(:,:,2)**
***[Flow]***

| *from:* / *to:* | Calcarine | DRN | MPFC | Amygdala |
|---|---|---|---|---|
| Calcarine | 0 | 0 | 0 | 0 |
| DRN | 0 | 0 | 1 | 0 |
| MPFC | 0 | 0 | 0 | 0 |
| Amygdala | 0 | 1 | 1 | 0 |

**DCM.b(:,:,3)**
***[Overload]***

| *from:* / *to:* | Calcarine | DRN | MPFC | Amygdala |
|---|---|---|---|---|
| Calcarine | 0 | 0 | 0 | 0 |
| DRN | 0 | 0 | 1 | 0 |
| MPFC | 0 | 0 | 0 | 0 |
| Amygdala | 0 | 1 | 1 | 0 |

**DCM.c**

| | Boredom | Flow | Overload |
|---|---|---|---|
| Calcarine | 1 | 1 | 1 |
| DRN | 0 | 0 | 0 |
| MPFC | 0 | 0 | 0 |
| Amygdala | 0 | 0 | 0 |

## Model 23 (member of family 1)

**DCM.a**

| from: / to: | Calcarine | DRN | MPFC | Amygdala |
|---|---|---|---|---|
| Calcarine | 1 | 1 | 1 | 1 |
| DRN | 1 | 1 | 1 | 1 |
| MPFC | 1 | 1 | 1 | 1 |
| Amygdala | 1 | 1 | 1 | 1 |

**DCM.b(:,:,1)**
***[Boredom]***

| from: / to: | Calcarine | DRN | MPFC | Amygdala |
|---|---|---|---|---|
| Calcarine | 0 | 0 | 0 | 0 |
| DRN | 0 | 0 | 1 | 1 |
| MPFC | 0 | 0 | 0 | 1 |
| Amygdala | 0 | 1 | 0 | 0 |

**DCM.b(:,:,2)**
***[Flow]***

| from: / to: | Calcarine | DRN | MPFC | Amygdala |
|---|---|---|---|---|
| Calcarine | 0 | 0 | 0 | 0 |
| DRN | 0 | 0 | 1 | 1 |
| MPFC | 0 | 0 | 0 | 1 |
| Amygdala | 0 | 1 | 0 | 0 |

**DCM.b(:,:,3)**
***[Overload]***

| from: / to: | Calcarine | DRN | MPFC | Amygdala |
|---|---|---|---|---|
| Calcarine | 0 | 0 | 0 | 0 |
| DRN | 0 | 0 | 1 | 1 |
| MPFC | 0 | 0 | 0 | 1 |
| Amygdala | 0 | 1 | 0 | 0 |

**DCM.c**

| | Boredom | Flow | Overload |
|---|---|---|---|
| Calcarine | 1 | 1 | 1 |
| DRN | 0 | 0 | 0 |
| MPFC | 0 | 0 | 0 |
| Amygdala | 0 | 0 | 0 |

## Model 24 (member of family 1)

**DCM.a**

| to: \ from: | Calcarine | DRN | MPFC | Amygdala |
|---|---|---|---|---|
| Calcarine | 1 | 1 | 1 | 1 |
| DRN | 1 | 1 | 1 | 1 |
| MPFC | 1 | 1 | 1 | 1 |
| Amygdala | 1 | 1 | 1 | 1 |

**DCM.b(:,:,1)**
***[Boredom]***

| to: \ from: | Calcarine | DRN | MPFC | Amygdala |
|---|---|---|---|---|
| Calcarine | 0 | 0 | 0 | 0 |
| DRN | 0 | 0 | 1 | 0 |
| MPFC | 0 | 0 | 0 | 1 |
| Amygdala | 0 | 1 | 0 | 0 |

**DCM.b(:,:,2)**
***[Flow]***

| to: \ from: | Calcarine | DRN | MPFC | Amygdala |
|---|---|---|---|---|
| Calcarine | 0 | 0 | 0 | 0 |
| DRN | 0 | 0 | 1 | 0 |
| MPFC | 0 | 0 | 0 | 1 |
| Amygdala | 0 | 1 | 0 | 0 |

**DCM.b(:,:,3)**
***[Overload]***

| to: \ from: | Calcarine | DRN | MPFC | Amygdala |
|---|---|---|---|---|
| Calcarine | 0 | 0 | 0 | 0 |
| DRN | 0 | 0 | 1 | 0 |
| MPFC | 0 | 0 | 0 | 1 |
| Amygdala | 0 | 1 | 0 | 0 |

**DCM.c**

| | Boredom | Flow | Overload |
|---|---|---|---|
| Calcarine | 1 | 1 | 1 |
| DRN | 0 | 0 | 0 |
| MPFC | 0 | 0 | 0 |
| Amygdala | 0 | 0 | 0 |

## Model 25 (member of family 1)

**DCM.a**

| to: \ from: | Calcarine | DRN | MPFC | Amygdala |
|---|---|---|---|---|
| Calcarine | 1 | 1 | 1 | 1 |
| DRN | 1 | 1 | 1 | 1 |
| MPFC | 1 | 1 | 1 | 1 |
| Amygdala | 1 | 1 | 1 | 1 |

**DCM.b(:,:,1)**
***[Boredom]***

| to: \ from: | Calcarine | DRN | MPFC | Amygdala |
|---|---|---|---|---|
| Calcarine | 0 | 0 | 0 | 0 |
| DRN | 0 | 0 | 1 | 1 |
| MPFC | 0 | 0 | 0 | 1 |
| Amygdala | 0 | 0 | 1 | 0 |

**DCM.b(:,:,2)**
***[Flow]***

| to: \ from: | Calcarine | DRN | MPFC | Amygdala |
|---|---|---|---|---|
| Calcarine | 0 | 0 | 0 | 0 |
| DRN | 0 | 0 | 1 | 1 |
| MPFC | 0 | 0 | 0 | 1 |
| Amygdala | 0 | 0 | 1 | 0 |

**DCM.b(:,:,3)**
***[Overload]***

| to: \ from: | Calcarine | DRN | MPFC | Amygdala |
|---|---|---|---|---|
| Calcarine | 0 | 0 | 0 | 0 |
| DRN | 0 | 0 | 1 | 1 |
| MPFC | 0 | 0 | 0 | 1 |
| Amygdala | 0 | 0 | 1 | 0 |

**DCM.c**

| | Boredom | Flow | Overload |
|---|---|---|---|
| Calcarine | 1 | 1 | 1 |
| DRN | 0 | 0 | 0 |
| MPFC | 0 | 0 | 0 |
| Amygdala | 0 | 0 | 0 |

## Model 26 (member of family 1)

**DCM.a**

| *from:* / *to:* | Calcarine | DRN | MPFC | Amygdala |
|---|---|---|---|---|
| Calcarine | 1 | 1 | 1 | 1 |
| DRN | 1 | 1 | 1 | 1 |
| MPFC | 1 | 1 | 1 | 1 |
| Amygdala | 1 | 1 | 1 | 1 |

**DCM.b(:,:,1)**
***[Boredom]***

| *from:* / *to:* | Calcarine | DRN | MPFC | Amygdala |
|---|---|---|---|---|
| Calcarine | 0 | 0 | 0 | 0 |
| DRN | 0 | 0 | 1 | 1 |
| MPFC | 0 | 0 | 0 | 0 |
| Amygdala | 0 | 0 | 1 | 0 |

**DCM.b(:,:,2)**
***[Flow]***

| *from:* / *to:* | Calcarine | DRN | MPFC | Amygdala |
|---|---|---|---|---|
| Calcarine | 0 | 0 | 0 | 0 |
| DRN | 0 | 0 | 1 | 1 |
| MPFC | 0 | 0 | 0 | 0 |
| Amygdala | 0 | 0 | 1 | 0 |

**DCM.b(:,:,3)**
***[Overload]***

| *from:* / *to:* | Calcarine | DRN | MPFC | Amygdala |
|---|---|---|---|---|
| Calcarine | 0 | 0 | 0 | 0 |
| DRN | 0 | 0 | 1 | 1 |
| MPFC | 0 | 0 | 0 | 0 |
| Amygdala | 0 | 0 | 1 | 0 |

**DCM.c**

| | Boredom | Flow | Overload |
|---|---|---|---|
| Calcarine | 1 | 1 | 1 |
| DRN | 0 | 0 | 0 |
| MPFC | 0 | 0 | 0 |
| Amygdala | 0 | 0 | 0 |

## Model 27 (member of family 1)

**DCM.a**

| from: / to: | Calcarine | DRN | MPFC | Amygdala |
|---|---|---|---|---|
| Calcarine | 1 | 1 | 1 | 1 |
| DRN | 1 | 1 | 1 | 1 |
| MPFC | 1 | 1 | 1 | 1 |
| Amygdala | 1 | 1 | 1 | 1 |

**DCM.b(:,:,1)**
***[Boredom]***

| from: / to: | Calcarine | DRN | MPFC | Amygdala |
|---|---|---|---|---|
| Calcarine | 0 | 0 | 0 | 0 |
| DRN | 0 | 0 | 1 | 1 |
| MPFC | 0 | 0 | 0 | 1 |
| Amygdala | 0 | 0 | 0 | 0 |

**DCM.b(:,:,2)**
***[Flow]***

| from: / to: | Calcarine | DRN | MPFC | Amygdala |
|---|---|---|---|---|
| Calcarine | 0 | 0 | 0 | 0 |
| DRN | 0 | 0 | 1 | 1 |
| MPFC | 0 | 0 | 0 | 1 |
| Amygdala | 0 | 0 | 0 | 0 |

**DCM.b(:,:,3)**
***[Overload]***

| from: / to: | Calcarine | DRN | MPFC | Amygdala |
|---|---|---|---|---|
| Calcarine | 0 | 0 | 0 | 0 |
| DRN | 0 | 0 | 1 | 1 |
| MPFC | 0 | 0 | 0 | 1 |
| Amygdala | 0 | 0 | 0 | 0 |

**DCM.c**

| | Boredom | Flow | Overload |
|---|---|---|---|
| Calcarine | 1 | 1 | 1 |
| DRN | 0 | 0 | 0 |
| MPFC | 0 | 0 | 0 |
| Amygdala | 0 | 0 | 0 |

## Model 28 (member of family 2)

**DCM.a**

| *to:* \ *from:* | Calcarine | DRN | MPFC | Amygdala |
|---|---|---|---|---|
| Calcarine | 1 | 1 | 1 | 1 |
| DRN | 1 | 1 | 1 | 1 |
| MPFC | 1 | 1 | 1 | 1 |
| Amygdala | 1 | 1 | 1 | 1 |

**DCM.b(:,:,1)**
***[Boredom]***

| *to:* \ *from:* | Calcarine | DRN | MPFC | Amygdala |
|---|---|---|---|---|
| Calcarine | 0 | 0 | 0 | 0 |
| DRN | 0 | 0 | 1 | 1 |
| MPFC | 0 | 1 | 0 | 0 |
| Amygdala | 0 | 1 | 0 | 0 |

**DCM.b(:,:,2)**
***[Flow]***

| *to:* \ *from:* | Calcarine | DRN | MPFC | Amygdala |
|---|---|---|---|---|
| Calcarine | 0 | 0 | 0 | 0 |
| DRN | 0 | 0 | 1 | 1 |
| MPFC | 0 | 1 | 0 | 0 |
| Amygdala | 0 | 1 | 0 | 0 |

**DCM.b(:,:,3)**
***[Overload]***

| *to:* \ *from:* | Calcarine | DRN | MPFC | Amygdala |
|---|---|---|---|---|
| Calcarine | 0 | 0 | 0 | 0 |
| DRN | 0 | 0 | 1 | 1 |
| MPFC | 0 | 1 | 0 | 0 |
| Amygdala | 0 | 1 | 0 | 0 |

**DCM.c**

| | Boredom | Flow | Overload |
|---|---|---|---|
| Calcarine | 1 | 1 | 1 |
| DRN | 0 | 0 | 0 |
| MPFC | 0 | 0 | 0 |
| Amygdala | 0 | 0 | 0 |

## Model 29 (member of family 2)

**DCM.a**

| to: \ from: | Calcarine | DRN | MPFC | Amygdala |
|---|---|---|---|---|
| Calcarine | 1 | 1 | 1 | 1 |
| DRN | 1 | 1 | 1 | 1 |
| MPFC | 1 | 1 | 1 | 1 |
| Amygdala | 1 | 1 | 1 | 1 |

**DCM.b(:,:,1)**
***[Boredom]***

| to: \ from: | Calcarine | DRN | MPFC | Amygdala |
|---|---|---|---|---|
| Calcarine | 0 | 0 | 0 | 0 |
| DRN | 0 | 0 | 1 | 0 |
| MPFC | 0 | 1 | 0 | 0 |
| Amygdala | 0 | 1 | 0 | 0 |

**DCM.b(:,:,2)**
***[Flow]***

| to: \ from: | Calcarine | DRN | MPFC | Amygdala |
|---|---|---|---|---|
| Calcarine | 0 | 0 | 0 | 0 |
| DRN | 0 | 0 | 1 | 0 |
| MPFC | 0 | 1 | 0 | 0 |
| Amygdala | 0 | 1 | 0 | 0 |

**DCM.b(:,:,3)**
***[Overload]***

| to: \ from: | Calcarine | DRN | MPFC | Amygdala |
|---|---|---|---|---|
| Calcarine | 0 | 0 | 0 | 0 |
| DRN | 0 | 0 | 1 | 0 |
| MPFC | 0 | 1 | 0 | 0 |
| Amygdala | 0 | 1 | 0 | 0 |

**DCM.c**

| | Boredom | Flow | Overload |
|---|---|---|---|
| Calcarine | 1 | 1 | 1 |
| DRN | 0 | 0 | 0 |
| MPFC | 0 | 0 | 0 |
| Amygdala | 0 | 0 | 0 |

## Model 30 (member of family 2)

**DCM.a**

| to: \ from: | Calcarine | DRN | MPFC | Amygdala |
|---|---|---|---|---|
| Calcarine | 1 | 1 | 1 | 1 |
| DRN | 1 | 1 | 1 | 1 |
| MPFC | 1 | 1 | 1 | 1 |
| Amygdala | 1 | 1 | 1 | 1 |

**DCM.b(:,:,1)**
***[Boredom]***

| to: \ from: | Calcarine | DRN | MPFC | Amygdala |
|---|---|---|---|---|
| Calcarine | 0 | 0 | 0 | 0 |
| DRN | 0 | 0 | 0 | 1 |
| MPFC | 0 | 1 | 0 | 0 |
| Amygdala | 0 | 1 | 0 | 0 |

**DCM.b(:,:,2)**
***[Flow]***

| to: \ from: | Calcarine | DRN | MPFC | Amygdala |
|---|---|---|---|---|
| Calcarine | 0 | 0 | 0 | 0 |
| DRN | 0 | 0 | 0 | 1 |
| MPFC | 0 | 1 | 0 | 0 |
| Amygdala | 0 | 1 | 0 | 0 |

**DCM.b(:,:,3)**
***[Overload]***

| to: \ from: | Calcarine | DRN | MPFC | Amygdala |
|---|---|---|---|---|
| Calcarine | 0 | 0 | 0 | 0 |
| DRN | 0 | 0 | 0 | 1 |
| MPFC | 0 | 1 | 0 | 0 |
| Amygdala | 0 | 1 | 0 | 0 |

**DCM.c**

| | Boredom | Flow | Overload |
|---|---|---|---|
| Calcarine | 1 | 1 | 1 |
| DRN | 0 | 0 | 0 |
| MPFC | 0 | 0 | 0 |
| Amygdala | 0 | 0 | 0 |

## Model 31 (member of family 2)

**DCM.a**

| from: / to: | Calcarine | DRN | MPFC | Amygdala |
|---|---|---|---|---|
| Calcarine | 1 | 1 | 1 | 1 |
| DRN | 1 | 1 | 1 | 1 |
| MPFC | 1 | 1 | 1 | 1 |
| Amygdala | 1 | 1 | 1 | 1 |

**DCM.b(:,:,1)**
***[Boredom]***

| from: / to: | Calcarine | DRN | MPFC | Amygdala |
|---|---|---|---|---|
| Calcarine | 0 | 0 | 0 | 0 |
| DRN | 0 | 0 | 0 | 0 |
| MPFC | 0 | 1 | 0 | 0 |
| Amygdala | 0 | 1 | 0 | 0 |

**DCM.b(:,:,2)**
***[Flow]***

| from: / to: | Calcarine | DRN | MPFC | Amygdala |
|---|---|---|---|---|
| Calcarine | 0 | 0 | 0 | 0 |
| DRN | 0 | 0 | 0 | 0 |
| MPFC | 0 | 1 | 0 | 0 |
| Amygdala | 0 | 1 | 0 | 0 |

**DCM.b(:,:,3)**
***[Overload]***

| from: / to: | Calcarine | DRN | MPFC | Amygdala |
|---|---|---|---|---|
| Calcarine | 0 | 0 | 0 | 0 |
| DRN | 0 | 0 | 0 | 0 |
| MPFC | 0 | 1 | 0 | 0 |
| Amygdala | 0 | 1 | 0 | 0 |

**DCM.c**

| | Boredom | Flow | Overload |
|---|---|---|---|
| Calcarine | 1 | 1 | 1 |
| DRN | 0 | 0 | 0 |
| MPFC | 0 | 0 | 0 |
| Amygdala | 0 | 0 | 0 |

## Model 32 (member of family 2)

**DCM.a**

| to: \ from: | Calcarine | DRN | MPFC | Amygdala |
|---|---|---|---|---|
| Calcarine | 1 | 1 | 1 | 1 |
| DRN | 1 | 1 | 1 | 1 |
| MPFC | 1 | 1 | 1 | 1 |
| Amygdala | 1 | 1 | 1 | 1 |

**DCM.b(:,:,1)**
***[Boredom]***

| to: \ from: | Calcarine | DRN | MPFC | Amygdala |
|---|---|---|---|---|
| Calcarine | 0 | 0 | 0 | 0 |
| DRN | 0 | 0 | 1 | 1 |
| MPFC | 0 | 1 | 0 | 0 |
| Amygdala | 0 | 0 | 0 | 0 |

**DCM.b(:,:,2)**
***[Flow]***

| to: \ from: | Calcarine | DRN | MPFC | Amygdala |
|---|---|---|---|---|
| Calcarine | 0 | 0 | 0 | 0 |
| DRN | 0 | 0 | 1 | 1 |
| MPFC | 0 | 1 | 0 | 0 |
| Amygdala | 0 | 0 | 0 | 0 |

**DCM.b(:,:,3)**
***[Overload]***

| to: \ from: | Calcarine | DRN | MPFC | Amygdala |
|---|---|---|---|---|
| Calcarine | 0 | 0 | 0 | 0 |
| DRN | 0 | 0 | 1 | 1 |
| MPFC | 0 | 1 | 0 | 0 |
| Amygdala | 0 | 0 | 0 | 0 |

**DCM.c**

| | Boredom | Flow | Overload |
|---|---|---|---|
| Calcarine | 1 | 1 | 1 |
| DRN | 0 | 0 | 0 |
| MPFC | 0 | 0 | 0 |
| Amygdala | 0 | 0 | 0 |

## Model 33 (member of family 2)

**DCM.a**

| *from:* / *to:* | Calcarine | DRN | MPFC | Amygdala |
|---|---|---|---|---|
| Calcarine | 1 | 1 | 1 | 1 |
| DRN | 1 | 1 | 1 | 1 |
| MPFC | 1 | 1 | 1 | 1 |
| Amygdala | 1 | 1 | 1 | 1 |

**DCM.b(:,:,1)**
***[Boredom]***

| *from:* / *to:* | Calcarine | DRN | MPFC | Amygdala |
|---|---|---|---|---|
| Calcarine | 0 | 0 | 0 | 0 |
| DRN | 0 | 0 | 0 | 1 |
| MPFC | 0 | 1 | 0 | 0 |
| Amygdala | 0 | 0 | 0 | 0 |

**DCM.b(:,:,2)**
***[Flow]***

| *from:* / *to:* | Calcarine | DRN | MPFC | Amygdala |
|---|---|---|---|---|
| Calcarine | 0 | 0 | 0 | 0 |
| DRN | 0 | 0 | 0 | 1 |
| MPFC | 0 | 1 | 0 | 0 |
| Amygdala | 0 | 0 | 0 | 0 |

**DCM.b(:,:,3)**
***[Overload]***

| *from:* / *to:* | Calcarine | DRN | MPFC | Amygdala |
|---|---|---|---|---|
| Calcarine | 0 | 0 | 0 | 0 |
| DRN | 0 | 0 | 0 | 1 |
| MPFC | 0 | 1 | 0 | 0 |
| Amygdala | 0 | 0 | 0 | 0 |

**DCM.c**

| | Boredom | Flow | Overload |
|---|---|---|---|
| Calcarine | 1 | 1 | 1 |
| DRN | 0 | 0 | 0 |
| MPFC | 0 | 0 | 0 |
| Amygdala | 0 | 0 | 0 |

## Model 34 (member of family 2)

**DCM.a**

| to: \ from: | Calcarine | DRN | MPFC | Amygdala |
|---|---|---|---|---|
| Calcarine | 1 | 1 | 1 | 1 |
| DRN | 1 | 1 | 1 | 1 |
| MPFC | 1 | 1 | 1 | 1 |
| Amygdala | 1 | 1 | 1 | 1 |

**DCM.b(:,:,1)**
***[Boredom]***

| to: \ from: | Calcarine | DRN | MPFC | Amygdala |
|---|---|---|---|---|
| Calcarine | 0 | 0 | 0 | 0 |
| DRN | 0 | 0 | 1 | 1 |
| MPFC | 0 | 0 | 0 | 0 |
| Amygdala | 0 | 1 | 0 | 0 |

**DCM.b(:,:,2)**
***[Flow]***

| to: \ from: | Calcarine | DRN | MPFC | Amygdala |
|---|---|---|---|---|
| Calcarine | 0 | 0 | 0 | 0 |
| DRN | 0 | 0 | 1 | 1 |
| MPFC | 0 | 0 | 0 | 0 |
| Amygdala | 0 | 1 | 0 | 0 |

**DCM.b(:,:,3)**
***[Overload]***

| to: \ from: | Calcarine | DRN | MPFC | Amygdala |
|---|---|---|---|---|
| Calcarine | 0 | 0 | 0 | 0 |
| DRN | 0 | 0 | 1 | 1 |
| MPFC | 0 | 0 | 0 | 0 |
| Amygdala | 0 | 1 | 0 | 0 |

**DCM.c**

| | Boredom | Flow | Overload |
|---|---|---|---|
| Calcarine | 1 | 1 | 1 |
| DRN | 0 | 0 | 0 |
| MPFC | 0 | 0 | 0 |
| Amygdala | 0 | 0 | 0 |

## Model 35 (member of family 2)

**DCM.a**

| to: \ from: | Calcarine | DRN | MPFC | Amygdala |
|---|---|---|---|---|
| Calcarine | 1 | 1 | 1 | 1 |
| DRN | 1 | 1 | 1 | 1 |
| MPFC | 1 | 1 | 1 | 1 |
| Amygdala | 1 | 1 | 1 | 1 |

**DCM.b(:,:,1)**
***[Boredom]***

| to: \ from: | Calcarine | DRN | MPFC | Amygdala |
|---|---|---|---|---|
| Calcarine | 0 | 0 | 0 | 0 |
| DRN | 0 | 0 | 1 | 0 |
| MPFC | 0 | 0 | 0 | 0 |
| Amygdala | 0 | 1 | 0 | 0 |

**DCM.b(:,:,2)**
***[Flow]***

| to: \ from: | Calcarine | DRN | MPFC | Amygdala |
|---|---|---|---|---|
| Calcarine | 0 | 0 | 0 | 0 |
| DRN | 0 | 0 | 1 | 0 |
| MPFC | 0 | 0 | 0 | 0 |
| Amygdala | 0 | 1 | 0 | 0 |

**DCM.b(:,:,3)**
***[Overload]***

| to: \ from: | Calcarine | DRN | MPFC | Amygdala |
|---|---|---|---|---|
| Calcarine | 0 | 0 | 0 | 0 |
| DRN | 0 | 0 | 1 | 0 |
| MPFC | 0 | 0 | 0 | 0 |
| Amygdala | 0 | 1 | 0 | 0 |

**DCM.c**

| | Boredom | Flow | Overload |
|---|---|---|---|
| Calcarine | 1 | 1 | 1 |
| DRN | 0 | 0 | 0 |
| MPFC | 0 | 0 | 0 |
| Amygdala | 0 | 0 | 0 |

## Model 36 (member of family 2)

**DCM.a**

| *to:* \ *from:* | Calcarine | DRN | MPFC | Amygdala |
|---|---|---|---|---|
| Calcarine | 1 | 1 | 1 | 1 |
| DRN | 1 | 1 | 1 | 1 |
| MPFC | 1 | 1 | 1 | 1 |
| Amygdala | 1 | 1 | 1 | 1 |

**DCM.b(:,:,1)**
***[Boredom]***

| *to:* \ *from:* | Calcarine | DRN | MPFC | Amygdala |
|---|---|---|---|---|
| Calcarine | 0 | 0 | 0 | 0 |
| DRN | 0 | 0 | 1 | 1 |
| MPFC | 0 | 0 | 0 | 0 |
| Amygdala | 0 | 0 | 0 | 0 |

**DCM.b(:,:,2)**
***[Flow]***

| *to:* \ *from:* | Calcarine | DRN | MPFC | Amygdala |
|---|---|---|---|---|
| Calcarine | 0 | 0 | 0 | 0 |
| DRN | 0 | 0 | 1 | 1 |
| MPFC | 0 | 0 | 0 | 0 |
| Amygdala | 0 | 0 | 0 | 0 |

**DCM.b(:,:,3)**
***[Overload]***

| *to:* \ *from:* | Calcarine | DRN | MPFC | Amygdala |
|---|---|---|---|---|
| Calcarine | 0 | 0 | 0 | 0 |
| DRN | 0 | 0 | 1 | 1 |
| MPFC | 0 | 0 | 0 | 0 |
| Amygdala | 0 | 0 | 0 | 0 |

**DCM.c**

| | Boredom | Flow | Overload |
|---|---|---|---|
| Calcarine | 1 | 1 | 1 |
| DRN | 0 | 0 | 0 |
| MPFC | 0 | 0 | 0 |
| Amygdala | 0 | 0 | 0 |

## Model 37 (member of family 3)

**DCM.a**

| from: / to: | Calcarine | DRN | MPFC | Amygdala |
|---|---|---|---|---|
| Calcarine | 1 | 1 | 1 | 1 |
| DRN | 1 | 1 | 1 | 1 |
| MPFC | 1 | 1 | 1 | 1 |
| Amygdala | 1 | 1 | 1 | 1 |

**DCM.b(:,:,1)**
***[Boredom]***

| from: / to: | Calcarine | DRN | MPFC | Amygdala |
|---|---|---|---|---|
| Calcarine | 0 | 0 | 0 | 0 |
| DRN | 0 | 0 | 1 | 0 |
| MPFC | 0 | 1 | 0 | 1 |
| Amygdala | 0 | 0 | 1 | 0 |

**DCM.b(:,:,2)**
***[Flow]***

| from: / to: | Calcarine | DRN | MPFC | Amygdala |
|---|---|---|---|---|
| Calcarine | 0 | 0 | 0 | 0 |
| DRN | 0 | 0 | 1 | 0 |
| MPFC | 0 | 1 | 0 | 1 |
| Amygdala | 0 | 0 | 1 | 0 |

**DCM.b(:,:,3)**
***[Overload]***

| from: / to: | Calcarine | DRN | MPFC | Amygdala |
|---|---|---|---|---|
| Calcarine | 0 | 0 | 0 | 0 |
| DRN | 0 | 0 | 1 | 0 |
| MPFC | 0 | 1 | 0 | 1 |
| Amygdala | 0 | 0 | 1 | 0 |

**DCM.c**

| | Boredom | Flow | Overload |
|---|---|---|---|
| Calcarine | 1 | 1 | 1 |
| DRN | 0 | 0 | 0 |
| MPFC | 0 | 0 | 0 |
| Amygdala | 0 | 0 | 0 |

## Model 38 (member of family 3)

**DCM.a**

| to: \ from: | Calcarine | DRN | MPFC | Amygdala |
|---|---|---|---|---|
| Calcarine | 1 | 1 | 1 | 1 |
| DRN | 1 | 1 | 1 | 1 |
| MPFC | 1 | 1 | 1 | 1 |
| Amygdala | 1 | 1 | 1 | 1 |

**DCM.b(:,:,1)**
***[Boredom]***

| to: \ from: | Calcarine | DRN | MPFC | Amygdala |
|---|---|---|---|---|
| Calcarine | 0 | 0 | 0 | 0 |
| DRN | 0 | 0 | 1 | 0 |
| MPFC | 0 | 1 | 0 | 0 |
| Amygdala | 0 | 0 | 1 | 0 |

**DCM.b(:,:,2)**
***[Flow]***

| to: \ from: | Calcarine | DRN | MPFC | Amygdala |
|---|---|---|---|---|
| Calcarine | 0 | 0 | 0 | 0 |
| DRN | 0 | 0 | 1 | 0 |
| MPFC | 0 | 1 | 0 | 0 |
| Amygdala | 0 | 0 | 1 | 0 |

**DCM.b(:,:,3)**
***[Overload]***

| to: \ from: | Calcarine | DRN | MPFC | Amygdala |
|---|---|---|---|---|
| Calcarine | 0 | 0 | 0 | 0 |
| DRN | 0 | 0 | 1 | 0 |
| MPFC | 0 | 1 | 0 | 0 |
| Amygdala | 0 | 0 | 1 | 0 |

**DCM.c**

| | Boredom | Flow | Overload |
|---|---|---|---|
| Calcarine | 1 | 1 | 1 |
| DRN | 0 | 0 | 0 |
| MPFC | 0 | 0 | 0 |
| Amygdala | 0 | 0 | 0 |

## Model 39 (member of family 3)

**DCM.a**

| to: \ from: | Calcarine | DRN | MPFC | Amygdala |
|---|---|---|---|---|
| Calcarine | 1 | 1 | 1 | 1 |
| DRN | 1 | 1 | 1 | 1 |
| MPFC | 1 | 1 | 1 | 1 |
| Amygdala | 1 | 1 | 1 | 1 |

**DCM.b(:,:,1)**
***[Boredom]***

| to: \ from: | Calcarine | DRN | MPFC | Amygdala |
|---|---|---|---|---|
| Calcarine | 0 | 0 | 0 | 0 |
| DRN | 0 | 0 | 1 | 0 |
| MPFC | 0 | 1 | 0 | 1 |
| Amygdala | 0 | 0 | 0 | 0 |

**DCM.b(:,:,2)**
***[Flow]***

| to: \ from: | Calcarine | DRN | MPFC | Amygdala |
|---|---|---|---|---|
| Calcarine | 0 | 0 | 0 | 0 |
| DRN | 0 | 0 | 1 | 0 |
| MPFC | 0 | 1 | 0 | 1 |
| Amygdala | 0 | 0 | 0 | 0 |

**DCM.b(:,:,3)**
***[Overload]***

| to: \ from: | Calcarine | DRN | MPFC | Amygdala |
|---|---|---|---|---|
| Calcarine | 0 | 0 | 0 | 0 |
| DRN | 0 | 0 | 1 | 0 |
| MPFC | 0 | 1 | 0 | 1 |
| Amygdala | 0 | 0 | 0 | 0 |

**DCM.c**

| | Boredom | Flow | Overload |
|---|---|---|---|
| Calcarine | 1 | 1 | 1 |
| DRN | 0 | 0 | 0 |
| MPFC | 0 | 0 | 0 |
| Amygdala | 0 | 0 | 0 |

## Model 40 (member of family 3)

**DCM.a**

| from: / to: | Calcarine | DRN | MPFC | Amygdala |
|---|---|---|---|---|
| Calcarine | 1 | 1 | 1 | 1 |
| DRN | 1 | 1 | 1 | 1 |
| MPFC | 1 | 1 | 1 | 1 |
| Amygdala | 1 | 1 | 1 | 1 |

**DCM.b(:,:,1)**
***[Boredom]***

| from: / to: | Calcarine | DRN | MPFC | Amygdala |
|---|---|---|---|---|
| Calcarine | 0 | 0 | 0 | 0 |
| DRN | 0 | 0 | 0 | 0 |
| MPFC | 0 | 1 | 0 | 1 |
| Amygdala | 0 | 0 | 1 | 0 |

**DCM.b(:,:,2)**
***[Flow]***

| from: / to: | Calcarine | DRN | MPFC | Amygdala |
|---|---|---|---|---|
| Calcarine | 0 | 0 | 0 | 0 |
| DRN | 0 | 0 | 0 | 0 |
| MPFC | 0 | 1 | 0 | 1 |
| Amygdala | 0 | 0 | 1 | 0 |

**DCM.b(:,:,3)**
***[Overload]***

| from: / to: | Calcarine | DRN | MPFC | Amygdala |
|---|---|---|---|---|
| Calcarine | 0 | 0 | 0 | 0 |
| DRN | 0 | 0 | 0 | 0 |
| MPFC | 0 | 1 | 0 | 1 |
| Amygdala | 0 | 0 | 1 | 0 |

**DCM.c**

| | Boredom | Flow | Overload |
|---|---|---|---|
| Calcarine | 1 | 1 | 1 |
| DRN | 0 | 0 | 0 |
| MPFC | 0 | 0 | 0 |
| Amygdala | 0 | 0 | 0 |

## Model 41 (member of family 3)

**DCM.a**

| from: / to: | Calcarine | DRN | MPFC | Amygdala |
|---|---|---|---|---|
| Calcarine | 1 | 1 | 1 | 1 |
| DRN | 1 | 1 | 1 | 1 |
| MPFC | 1 | 1 | 1 | 1 |
| Amygdala | 1 | 1 | 1 | 1 |

**DCM.b(:,:,1)**
***[Boredom]***

| from: / to: | Calcarine | DRN | MPFC | Amygdala |
|---|---|---|---|---|
| Calcarine | 0 | 0 | 0 | 0 |
| DRN | 0 | 0 | 0 | 0 |
| MPFC | 0 | 1 | 0 | 0 |
| Amygdala | 0 | 0 | 1 | 0 |

**DCM.b(:,:,2)**
***[Flow]***

| from: / to: | Calcarine | DRN | MPFC | Amygdala |
|---|---|---|---|---|
| Calcarine | 0 | 0 | 0 | 0 |
| DRN | 0 | 0 | 0 | 0 |
| MPFC | 0 | 1 | 0 | 0 |
| Amygdala | 0 | 0 | 1 | 0 |

**DCM.b(:,:,3)**
***[Overload]***

| from: / to: | Calcarine | DRN | MPFC | Amygdala |
|---|---|---|---|---|
| Calcarine | 0 | 0 | 0 | 0 |
| DRN | 0 | 0 | 0 | 0 |
| MPFC | 0 | 1 | 0 | 0 |
| Amygdala | 0 | 0 | 1 | 0 |

**DCM.c**

| | Boredom | Flow | Overload |
|---|---|---|---|
| Calcarine | 1 | 1 | 1 |
| DRN | 0 | 0 | 0 |
| MPFC | 0 | 0 | 0 |
| Amygdala | 0 | 0 | 0 |

## Model 42 (member of family 3)

**DCM.a**

| to: \ from: | Calcarine | DRN | MPFC | Amygdala |
|---|---|---|---|---|
| Calcarine | 1 | 1 | 1 | 1 |
| DRN | 1 | 1 | 1 | 1 |
| MPFC | 1 | 1 | 1 | 1 |
| Amygdala | 1 | 1 | 1 | 1 |

**DCM.b(:,:,1)**
***[Boredom]***

| to: \ from: | Calcarine | DRN | MPFC | Amygdala |
|---|---|---|---|---|
| Calcarine | 0 | 0 | 0 | 0 |
| DRN | 0 | 0 | 0 | 0 |
| MPFC | 0 | 1 | 0 | 1 |
| Amygdala | 0 | 0 | 0 | 0 |

**DCM.b(:,:,2)**
***[Flow]***

| to: \ from: | Calcarine | DRN | MPFC | Amygdala |
|---|---|---|---|---|
| Calcarine | 0 | 0 | 0 | 0 |
| DRN | 0 | 0 | 0 | 0 |
| MPFC | 0 | 1 | 0 | 1 |
| Amygdala | 0 | 0 | 0 | 0 |

**DCM.b(:,:,3)**
***[Overload]***

| to: \ from: | Calcarine | DRN | MPFC | Amygdala |
|---|---|---|---|---|
| Calcarine | 0 | 0 | 0 | 0 |
| DRN | 0 | 0 | 0 | 0 |
| MPFC | 0 | 1 | 0 | 1 |
| Amygdala | 0 | 0 | 0 | 0 |

**DCM.c**

| | Boredom | Flow | Overload |
|---|---|---|---|
| Calcarine | 1 | 1 | 1 |
| DRN | 0 | 0 | 0 |
| MPFC | 0 | 0 | 0 |
| Amygdala | 0 | 0 | 0 |

## Model 43 (member of family 3)

**DCM.a**

| from: / to: | Calcarine | DRN | MPFC | Amygdala |
|---|---|---|---|---|
| Calcarine | 1 | 1 | 1 | 1 |
| DRN | 1 | 1 | 1 | 1 |
| MPFC | 1 | 1 | 1 | 1 |
| Amygdala | 1 | 1 | 1 | 1 |

**DCM.b(:,:,1)**
***[Boredom]***

| from: / to: | Calcarine | DRN | MPFC | Amygdala |
|---|---|---|---|---|
| Calcarine | 0 | 0 | 0 | 0 |
| DRN | 0 | 0 | 1 | 0 |
| MPFC | 0 | 0 | 0 | 1 |
| Amygdala | 0 | 0 | 1 | 0 |

**DCM.b(:,:,2)**
***[Flow]***

| from: / to: | Calcarine | DRN | MPFC | Amygdala |
|---|---|---|---|---|
| Calcarine | 0 | 0 | 0 | 0 |
| DRN | 0 | 0 | 1 | 0 |
| MPFC | 0 | 0 | 0 | 1 |
| Amygdala | 0 | 0 | 1 | 0 |

**DCM.b(:,:,3)**
***[Overload]***

| from: / to: | Calcarine | DRN | MPFC | Amygdala |
|---|---|---|---|---|
| Calcarine | 0 | 0 | 0 | 0 |
| DRN | 0 | 0 | 1 | 0 |
| MPFC | 0 | 0 | 0 | 1 |
| Amygdala | 0 | 0 | 1 | 0 |

**DCM.c**

| | Boredom | Flow | Overload |
|---|---|---|---|
| Calcarine | 1 | 1 | 1 |
| DRN | 0 | 0 | 0 |
| MPFC | 0 | 0 | 0 |
| Amygdala | 0 | 0 | 0 |

# Model 44 (member of family 3)

**DCM.a**

| to: \ from: | Calcarine | DRN | MPFC | Amygdala |
|---|---|---|---|---|
| Calcarine | 1 | 1 | 1 | 1 |
| DRN | 1 | 1 | 1 | 1 |
| MPFC | 1 | 1 | 1 | 1 |
| Amygdala | 1 | 1 | 1 | 1 |

**DCM.b(:,:,1)**
***[Boredom]***

| to: \ from: | Calcarine | DRN | MPFC | Amygdala |
|---|---|---|---|---|
| Calcarine | 0 | 0 | 0 | 0 |
| DRN | 0 | 0 | 1 | 0 |
| MPFC | 0 | 0 | 0 | 0 |
| Amygdala | 0 | 0 | 1 | 0 |

**DCM.b(:,:,2)**
***[Flow]***

| to: \ from: | Calcarine | DRN | MPFC | Amygdala |
|---|---|---|---|---|
| Calcarine | 0 | 0 | 0 | 0 |
| DRN | 0 | 0 | 1 | 0 |
| MPFC | 0 | 0 | 0 | 0 |
| Amygdala | 0 | 0 | 1 | 0 |

**DCM.b(:,:,3)**
***[Overload]***

| to: \ from: | Calcarine | DRN | MPFC | Amygdala |
|---|---|---|---|---|
| Calcarine | 0 | 0 | 0 | 0 |
| DRN | 0 | 0 | 1 | 0 |
| MPFC | 0 | 0 | 0 | 0 |
| Amygdala | 0 | 0 | 1 | 0 |

**DCM.c**

| | Boredom | Flow | Overload |
|---|---|---|---|
| Calcarine | 1 | 1 | 1 |
| DRN | 0 | 0 | 0 |
| MPFC | 0 | 0 | 0 |
| Amygdala | 0 | 0 | 0 |

## Model 45 (member of family 3)

**DCM.a**

| from: / to: | Calcarine | DRN | MPFC | Amygdala |
|---|---|---|---|---|
| Calcarine | 1 | 1 | 1 | 1 |
| DRN | 1 | 1 | 1 | 1 |
| MPFC | 1 | 1 | 1 | 1 |
| Amygdala | 1 | 1 | 1 | 1 |

**DCM.b(:,:,1)**
***[Boredom]***

| from: / to: | Calcarine | DRN | MPFC | Amygdala |
|---|---|---|---|---|
| Calcarine | 0 | 0 | 0 | 0 |
| DRN | 0 | 0 | 1 | 0 |
| MPFC | 0 | 0 | 0 | 1 |
| Amygdala | 0 | 0 | 0 | 0 |

**DCM.b(:,:,2)**
***[Flow]***

| from: / to: | Calcarine | DRN | MPFC | Amygdala |
|---|---|---|---|---|
| Calcarine | 0 | 0 | 0 | 0 |
| DRN | 0 | 0 | 1 | 0 |
| MPFC | 0 | 0 | 0 | 1 |
| Amygdala | 0 | 0 | 0 | 0 |

**DCM.b(:,:,3)**
***[Overload]***

| from: / to: | Calcarine | DRN | MPFC | Amygdala |
|---|---|---|---|---|
| Calcarine | 0 | 0 | 0 | 0 |
| DRN | 0 | 0 | 1 | 0 |
| MPFC | 0 | 0 | 0 | 1 |
| Amygdala | 0 | 0 | 0 | 0 |

**DCM.c**

| | Boredom | Flow | Overload |
|---|---|---|---|
| Calcarine | 1 | 1 | 1 |
| DRN | 0 | 0 | 0 |
| MPFC | 0 | 0 | 0 |
| Amygdala | 0 | 0 | 0 |

## Model 46 (member of family 4)

**DCM.a**

| from: / to: | Calcarine | DRN | MPFC | Amygdala |
|---|---|---|---|---|
| Calcarine | 1 | 1 | 1 | 1 |
| DRN | 1 | 1 | 1 | 1 |
| MPFC | 1 | 1 | 1 | 1 |
| Amygdala | 1 | 1 | 1 | 1 |

**DCM.b(:,:,1)**
***[Boredom]***

| from: / to: | Calcarine | DRN | MPFC | Amygdala |
|---|---|---|---|---|
| Calcarine | 0 | 0 | 0 | 0 |
| DRN | 0 | 0 | 0 | 1 |
| MPFC | 0 | 0 | 0 | 1 |
| Amygdala | 0 | 1 | 1 | 0 |

**DCM.b(:,:,2)**
***[Flow]***

| from: / to: | Calcarine | DRN | MPFC | Amygdala |
|---|---|---|---|---|
| Calcarine | 0 | 0 | 0 | 0 |
| DRN | 0 | 0 | 0 | 1 |
| MPFC | 0 | 0 | 0 | 1 |
| Amygdala | 0 | 1 | 1 | 0 |

**DCM.b(:,:,3)**
***[Overload]***

| from: / to: | Calcarine | DRN | MPFC | Amygdala |
|---|---|---|---|---|
| Calcarine | 0 | 0 | 0 | 0 |
| DRN | 0 | 0 | 0 | 1 |
| MPFC | 0 | 0 | 0 | 1 |
| Amygdala | 0 | 1 | 1 | 0 |

**DCM.c**

| | Boredom | Flow | Overload |
|---|---|---|---|
| Calcarine | 1 | 1 | 1 |
| DRN | 0 | 0 | 0 |
| MPFC | 0 | 0 | 0 |
| Amygdala | 0 | 0 | 0 |

## Model 47 (member of family 4)

**DCM.a**

| to: \ from: | Calcarine | DRN | MPFC | Amygdala |
|---|---|---|---|---|
| Calcarine | 1 | 1 | 1 | 1 |
| DRN | 1 | 1 | 1 | 1 |
| MPFC | 1 | 1 | 1 | 1 |
| Amygdala | 1 | 1 | 1 | 1 |

**DCM.b(:,:,1)**
***[Boredom]***

| to: \ from: | Calcarine | DRN | MPFC | Amygdala |
|---|---|---|---|---|
| Calcarine | 0 | 0 | 0 | 0 |
| DRN | 0 | 0 | 0 | 1 |
| MPFC | 0 | 0 | 0 | 0 |
| Amygdala | 0 | 1 | 1 | 0 |

**DCM.b(:,:,2)**
***[Flow]***

| to: \ from: | Calcarine | DRN | MPFC | Amygdala |
|---|---|---|---|---|
| Calcarine | 0 | 0 | 0 | 0 |
| DRN | 0 | 0 | 0 | 1 |
| MPFC | 0 | 0 | 0 | 0 |
| Amygdala | 0 | 1 | 1 | 0 |

**DCM.b(:,:,3)**
***[Overload]***

| to: \ from: | Calcarine | DRN | MPFC | Amygdala |
|---|---|---|---|---|
| Calcarine | 0 | 0 | 0 | 0 |
| DRN | 0 | 0 | 0 | 1 |
| MPFC | 0 | 0 | 0 | 0 |
| Amygdala | 0 | 1 | 1 | 0 |

**DCM.c**

| | Boredom | Flow | Overload |
|---|---|---|---|
| Calcarine | 1 | 1 | 1 |
| DRN | 0 | 0 | 0 |
| MPFC | 0 | 0 | 0 |
| Amygdala | 0 | 0 | 0 |

## Model 48 (member of family 4)

**DCM.a**

| from: / to: | Calcarine | DRN | MPFC | Amygdala |
|---|---|---|---|---|
| Calcarine | 1 | 1 | 1 | 1 |
| DRN | 1 | 1 | 1 | 1 |
| MPFC | 1 | 1 | 1 | 1 |
| Amygdala | 1 | 1 | 1 | 1 |

**DCM.b(:,:,1)**
***[Boredom]***

| from: / to: | Calcarine | DRN | MPFC | Amygdala |
|---|---|---|---|---|
| Calcarine | 0 | 0 | 0 | 0 |
| DRN | 0 | 0 | 0 | 0 |
| MPFC | 0 | 0 | 0 | 1 |
| Amygdala | 0 | 1 | 1 | 0 |

**DCM.b(:,:,2)**
***[Flow]***

| from: / to: | Calcarine | DRN | MPFC | Amygdala |
|---|---|---|---|---|
| Calcarine | 0 | 0 | 0 | 0 |
| DRN | 0 | 0 | 0 | 0 |
| MPFC | 0 | 0 | 0 | 1 |
| Amygdala | 0 | 1 | 1 | 0 |

**DCM.b(:,:,3)**
***[Overload]***

| from: / to: | Calcarine | DRN | MPFC | Amygdala |
|---|---|---|---|---|
| Calcarine | 0 | 0 | 0 | 0 |
| DRN | 0 | 0 | 0 | 0 |
| MPFC | 0 | 0 | 0 | 1 |
| Amygdala | 0 | 1 | 1 | 0 |

**DCM.c**

| | Boredom | Flow | Overload |
|---|---|---|---|
| Calcarine | 1 | 1 | 1 |
| DRN | 0 | 0 | 0 |
| MPFC | 0 | 0 | 0 |
| Amygdala | 0 | 0 | 0 |

## Model 49 (member of family 4)

**DCM.a**

| to: \ from: | Calcarine | DRN | MPFC | Amygdala |
|---|---|---|---|---|
| Calcarine | 1 | 1 | 1 | 1 |
| DRN | 1 | 1 | 1 | 1 |
| MPFC | 1 | 1 | 1 | 1 |
| Amygdala | 1 | 1 | 1 | 1 |

**DCM.b(:,:,1)**
***[Boredom]***

| to: \ from: | Calcarine | DRN | MPFC | Amygdala |
|---|---|---|---|---|
| Calcarine | 0 | 0 | 0 | 0 |
| DRN | 0 | 0 | 0 | 0 |
| MPFC | 0 | 0 | 0 | 0 |
| Amygdala | 0 | 1 | 1 | 0 |

**DCM.b(:,:,2)**
***[Flow]***

| to: \ from: | Calcarine | DRN | MPFC | Amygdala |
|---|---|---|---|---|
| Calcarine | 0 | 0 | 0 | 0 |
| DRN | 0 | 0 | 0 | 0 |
| MPFC | 0 | 0 | 0 | 0 |
| Amygdala | 0 | 1 | 1 | 0 |

**DCM.b(:,:,3)**
***[Overload]***

| to: \ from: | Calcarine | DRN | MPFC | Amygdala |
|---|---|---|---|---|
| Calcarine | 0 | 0 | 0 | 0 |
| DRN | 0 | 0 | 0 | 0 |
| MPFC | 0 | 0 | 0 | 0 |
| Amygdala | 0 | 1 | 1 | 0 |

**DCM.c**

| | Boredom | Flow | Overload |
|---|---|---|---|
| Calcarine | 1 | 1 | 1 |
| DRN | 0 | 0 | 0 |
| MPFC | 0 | 0 | 0 |
| Amygdala | 0 | 0 | 0 |

## Model 50 (member of family 4)

**DCM.a**

| *to:* \ *from:* | Calcarine | DRN | MPFC | Amygdala |
|---|---|---|---|---|
| Calcarine | 1 | 1 | 1 | 1 |
| DRN | 1 | 1 | 1 | 1 |
| MPFC | 1 | 1 | 1 | 1 |
| Amygdala | 1 | 1 | 1 | 1 |

**DCM.b(:,:,1)**
***[Boredom]***

| *to:* \ *from:* | Calcarine | DRN | MPFC | Amygdala |
|---|---|---|---|---|
| Calcarine | 0 | 0 | 0 | 0 |
| DRN | 0 | 0 | 0 | 1 |
| MPFC | 0 | 0 | 0 | 1 |
| Amygdala | 0 | 1 | 0 | 0 |

**DCM.b(:,:,2)**
***[Flow]***

| *to:* \ *from:* | Calcarine | DRN | MPFC | Amygdala |
|---|---|---|---|---|
| Calcarine | 0 | 0 | 0 | 0 |
| DRN | 0 | 0 | 0 | 1 |
| MPFC | 0 | 0 | 0 | 1 |
| Amygdala | 0 | 1 | 0 | 0 |

**DCM.b(:,:,3)**
***[Overload]***

| *to:* \ *from:* | Calcarine | DRN | MPFC | Amygdala |
|---|---|---|---|---|
| Calcarine | 0 | 0 | 0 | 0 |
| DRN | 0 | 0 | 0 | 1 |
| MPFC | 0 | 0 | 0 | 1 |
| Amygdala | 0 | 1 | 0 | 0 |

**DCM.c**

| | Boredom | Flow | Overload |
|---|---|---|---|
| Calcarine | 1 | 1 | 1 |
| DRN | 0 | 0 | 0 |
| MPFC | 0 | 0 | 0 |
| Amygdala | 0 | 0 | 0 |

## Model 51 (member of family 4)

**DCM.a**

| to: \ from: | Calcarine | DRN | MPFC | Amygdala |
|---|---|---|---|---|
| Calcarine | 1 | 1 | 1 | 1 |
| DRN | 1 | 1 | 1 | 1 |
| MPFC | 1 | 1 | 1 | 1 |
| Amygdala | 1 | 1 | 1 | 1 |

**DCM.b(:,:,1)**
***[Boredom]***

| to: \ from: | Calcarine | DRN | MPFC | Amygdala |
|---|---|---|---|---|
| Calcarine | 0 | 0 | 0 | 0 |
| DRN | 0 | 0 | 0 | 0 |
| MPFC | 0 | 0 | 0 | 1 |
| Amygdala | 0 | 1 | 0 | 0 |

**DCM.b(:,:,2)**
***[Flow]***

| to: \ from: | Calcarine | DRN | MPFC | Amygdala |
|---|---|---|---|---|
| Calcarine | 0 | 0 | 0 | 0 |
| DRN | 0 | 0 | 0 | 0 |
| MPFC | 0 | 0 | 0 | 1 |
| Amygdala | 0 | 1 | 0 | 0 |

**DCM.b(:,:,3)**
***[Overload]***

| to: \ from: | Calcarine | DRN | MPFC | Amygdala |
|---|---|---|---|---|
| Calcarine | 0 | 0 | 0 | 0 |
| DRN | 0 | 0 | 0 | 0 |
| MPFC | 0 | 0 | 0 | 1 |
| Amygdala | 0 | 1 | 0 | 0 |

**DCM.c**

| | Boredom | Flow | Overload |
|---|---|---|---|
| Calcarine | 1 | 1 | 1 |
| DRN | 0 | 0 | 0 |
| MPFC | 0 | 0 | 0 |
| Amygdala | 0 | 0 | 0 |

## Model 52 (member of family 4)

**DCM.a**

| from: / to: | Calcarine | DRN | MPFC | Amygdala |
|---|---|---|---|---|
| Calcarine | 1 | 1 | 1 | 1 |
| DRN | 1 | 1 | 1 | 1 |
| MPFC | 1 | 1 | 1 | 1 |
| Amygdala | 1 | 1 | 1 | 1 |

**DCM.b(:,:,1)**
***[Boredom]***

| from: / to: | Calcarine | DRN | MPFC | Amygdala |
|---|---|---|---|---|
| Calcarine | 0 | 0 | 0 | 0 |
| DRN | 0 | 0 | 0 | 1 |
| MPFC | 0 | 0 | 0 | 1 |
| Amygdala | 0 | 0 | 1 | 0 |

**DCM.b(:,:,2)**
***[Flow]***

| from: / to: | Calcarine | DRN | MPFC | Amygdala |
|---|---|---|---|---|
| Calcarine | 0 | 0 | 0 | 0 |
| DRN | 0 | 0 | 0 | 1 |
| MPFC | 0 | 0 | 0 | 1 |
| Amygdala | 0 | 0 | 1 | 0 |

**DCM.b(:,:,3)**
***[Overload]***

| from: / to: | Calcarine | DRN | MPFC | Amygdala |
|---|---|---|---|---|
| Calcarine | 0 | 0 | 0 | 0 |
| DRN | 0 | 0 | 0 | 1 |
| MPFC | 0 | 0 | 0 | 1 |
| Amygdala | 0 | 0 | 1 | 0 |

**DCM.c**

| | Boredom | Flow | Overload |
|---|---|---|---|
| Calcarine | 1 | 1 | 1 |
| DRN | 0 | 0 | 0 |
| MPFC | 0 | 0 | 0 |
| Amygdala | 0 | 0 | 0 |

## Model 53 (member of family 4)

**DCM.a**

| *from:* / *to:* | Calcarine | DRN | MPFC | Amygdala |
|---|---|---|---|---|
| Calcarine | 1 | 1 | 1 | 1 |
| DRN | 1 | 1 | 1 | 1 |
| MPFC | 1 | 1 | 1 | 1 |
| Amygdala | 1 | 1 | 1 | 1 |

**DCM.b(:,:,1)**
***[Boredom]***

| *from:* / *to:* | Calcarine | DRN | MPFC | Amygdala |
|---|---|---|---|---|
| Calcarine | 0 | 0 | 0 | 0 |
| DRN | 0 | 0 | 0 | 1 |
| MPFC | 0 | 0 | 0 | 0 |
| Amygdala | 0 | 0 | 1 | 0 |

**DCM.b(:,:,2)**
***[Flow]***

| *from:* / *to:* | Calcarine | DRN | MPFC | Amygdala |
|---|---|---|---|---|
| Calcarine | 0 | 0 | 0 | 0 |
| DRN | 0 | 0 | 0 | 1 |
| MPFC | 0 | 0 | 0 | 0 |
| Amygdala | 0 | 0 | 1 | 0 |

**DCM.b(:,:,3)**
***[Overload]***

| *from:* / *to:* | Calcarine | DRN | MPFC | Amygdala |
|---|---|---|---|---|
| Calcarine | 0 | 0 | 0 | 0 |
| DRN | 0 | 0 | 0 | 1 |
| MPFC | 0 | 0 | 0 | 0 |
| Amygdala | 0 | 0 | 1 | 0 |

**DCM.c**

| | Boredom | Flow | Overload |
|---|---|---|---|
| Calcarine | 1 | 1 | 1 |
| DRN | 0 | 0 | 0 |
| MPFC | 0 | 0 | 0 |
| Amygdala | 0 | 0 | 0 |

## Model 54 (member of family 4)

**DCM.a**

| from: / to: | Calcarine | DRN | MPFC | Amygdala |
|---|---|---|---|---|
| Calcarine | 1 | 1 | 1 | 1 |
| DRN | 1 | 1 | 1 | 1 |
| MPFC | 1 | 1 | 1 | 1 |
| Amygdala | 1 | 1 | 1 | 1 |

**DCM.b(:,:,1)**
***[Boredom]***

| from: / to: | Calcarine | DRN | MPFC | Amygdala |
|---|---|---|---|---|
| Calcarine | 0 | 0 | 0 | 0 |
| DRN | 0 | 0 | 0 | 1 |
| MPFC | 0 | 0 | 0 | 1 |
| Amygdala | 0 | 0 | 0 | 0 |

**DCM.b(:,:,2)**
***[Flow]***

| from: / to: | Calcarine | DRN | MPFC | Amygdala |
|---|---|---|---|---|
| Calcarine | 0 | 0 | 0 | 0 |
| DRN | 0 | 0 | 0 | 1 |
| MPFC | 0 | 0 | 0 | 1 |
| Amygdala | 0 | 0 | 0 | 0 |

**DCM.b(:,:,3)**
***[Overload]***

| from: / to: | Calcarine | DRN | MPFC | Amygdala |
|---|---|---|---|---|
| Calcarine | 0 | 0 | 0 | 0 |
| DRN | 0 | 0 | 0 | 1 |
| MPFC | 0 | 0 | 0 | 1 |
| Amygdala | 0 | 0 | 0 | 0 |

**DCM.c**

| | Boredom | Flow | Overload |
|---|---|---|---|
| Calcarine | 1 | 1 | 1 |
| DRN | 0 | 0 | 0 |
| MPFC | 0 | 0 | 0 |
| Amygdala | 0 | 0 | 0 |

## Model 55 (member of family 5)

**DCM.a**

| from: / to: | Calcarine | DRN | MPFC | Amygdala |
|---|---|---|---|---|
| Calcarine | 1 | 1 | 1 | 1 |
| DRN | 1 | 1 | 1 | 1 |
| MPFC | 1 | 1 | 1 | 1 |
| Amygdala | 1 | 1 | 1 | 1 |

**DCM.b(:,:,1)**
***[Boredom]***

| from: / to: | Calcarine | DRN | MPFC | Amygdala |
|---|---|---|---|---|
| Calcarine | 0 | 0 | 0 | 0 |
| DRN | 0 | 0 | 1 | 0 |
| MPFC | 0 | 1 | 0 | 0 |
| Amygdala | 0 | 0 | 0 | 0 |

**DCM.b(:,:,2)**
***[Flow]***

| from: / to: | Calcarine | DRN | MPFC | Amygdala |
|---|---|---|---|---|
| Calcarine | 0 | 0 | 0 | 0 |
| DRN | 0 | 0 | 1 | 0 |
| MPFC | 0 | 1 | 0 | 0 |
| Amygdala | 0 | 0 | 0 | 0 |

**DCM.b(:,:,3)**
***[Overload]***

| from: / to: | Calcarine | DRN | MPFC | Amygdala |
|---|---|---|---|---|
| Calcarine | 0 | 0 | 0 | 0 |
| DRN | 0 | 0 | 1 | 0 |
| MPFC | 0 | 1 | 0 | 0 |
| Amygdala | 0 | 0 | 0 | 0 |

**DCM.c**

| | Boredom | Flow | Overload |
|---|---|---|---|
| Calcarine | 1 | 1 | 1 |
| DRN | 0 | 0 | 0 |
| MPFC | 0 | 0 | 0 |
| Amygdala | 0 | 0 | 0 |

## Model 56 (member of family 5)

**DCM.a**

| to: \ from: | Calcarine | DRN | MPFC | Amygdala |
|---|---|---|---|---|
| Calcarine | 1 | 1 | 1 | 1 |
| DRN | 1 | 1 | 1 | 1 |
| MPFC | 1 | 1 | 1 | 1 |
| Amygdala | 1 | 1 | 1 | 1 |

**DCM.b(:,:,1)**
***[Boredom]***

| to: \ from: | Calcarine | DRN | MPFC | Amygdala |
|---|---|---|---|---|
| Calcarine | 0 | 0 | 0 | 0 |
| DRN | 0 | 0 | 0 | 0 |
| MPFC | 0 | 1 | 0 | 0 |
| Amygdala | 0 | 0 | 0 | 0 |

**DCM.b(:,:,2)**
***[Flow]***

| to: \ from: | Calcarine | DRN | MPFC | Amygdala |
|---|---|---|---|---|
| Calcarine | 0 | 0 | 0 | 0 |
| DRN | 0 | 0 | 0 | 0 |
| MPFC | 0 | 1 | 0 | 0 |
| Amygdala | 0 | 0 | 0 | 0 |

**DCM.b(:,:,3)**
***[Overload]***

| to: \ from: | Calcarine | DRN | MPFC | Amygdala |
|---|---|---|---|---|
| Calcarine | 0 | 0 | 0 | 0 |
| DRN | 0 | 0 | 0 | 0 |
| MPFC | 0 | 1 | 0 | 0 |
| Amygdala | 0 | 0 | 0 | 0 |

**DCM.c**

| | Boredom | Flow | Overload |
|---|---|---|---|
| Calcarine | 1 | 1 | 1 |
| DRN | 0 | 0 | 0 |
| MPFC | 0 | 0 | 0 |
| Amygdala | 0 | 0 | 0 |

## Model 57 (member of family 5)

**DCM.a**

| *from:* / *to:* | Calcarine | DRN | MPFC | Amygdala |
|---|---|---|---|---|
| Calcarine | 1 | 1 | 1 | 1 |
| DRN | 1 | 1 | 1 | 1 |
| MPFC | 1 | 1 | 1 | 1 |
| Amygdala | 1 | 1 | 1 | 1 |

**DCM.b(:,:,1)**
***[Boredom]***

| *from:* / *to:* | Calcarine | DRN | MPFC | Amygdala |
|---|---|---|---|---|
| Calcarine | 0 | 0 | 0 | 0 |
| DRN | 0 | 0 | 1 | 0 |
| MPFC | 0 | 0 | 0 | 0 |
| Amygdala | 0 | 0 | 0 | 0 |

**DCM.b(:,:,2)**
***[Flow]***

| *from:* / *to:* | Calcarine | DRN | MPFC | Amygdala |
|---|---|---|---|---|
| Calcarine | 0 | 0 | 0 | 0 |
| DRN | 0 | 0 | 1 | 0 |
| MPFC | 0 | 0 | 0 | 0 |
| Amygdala | 0 | 0 | 0 | 0 |

**DCM.b(:,:,3)**
***[Overload]***

| *from:* / *to:* | Calcarine | DRN | MPFC | Amygdala |
|---|---|---|---|---|
| Calcarine | 0 | 0 | 0 | 0 |
| DRN | 0 | 0 | 1 | 0 |
| MPFC | 0 | 0 | 0 | 0 |
| Amygdala | 0 | 0 | 0 | 0 |

**DCM.c**

| | Boredom | Flow | Overload |
|---|---|---|---|
| Calcarine | 1 | 1 | 1 |
| DRN | 0 | 0 | 0 |
| MPFC | 0 | 0 | 0 |
| Amygdala | 0 | 0 | 0 |

## Model 58 (member of family 6)

**DCM.a**

| *from:* / *to:* | Calcarine | DRN | MPFC | Amygdala |
|---|---|---|---|---|
| Calcarine | 1 | 1 | 1 | 1 |
| DRN | 1 | 1 | 1 | 1 |
| MPFC | 1 | 1 | 1 | 1 |
| Amygdala | 1 | 1 | 1 | 1 |

**DCM.b(:,:,1)**
***[Boredom]***

| *from:* / *to:* | Calcarine | DRN | MPFC | Amygdala |
|---|---|---|---|---|
| Calcarine | 0 | 0 | 0 | 0 |
| DRN | 0 | 0 | 0 | 1 |
| MPFC | 0 | 0 | 0 | 0 |
| Amygdala | 0 | 1 | 0 | 0 |

**DCM.b(:,:,2)**
***[Flow]***

| *from:* / *to:* | Calcarine | DRN | MPFC | Amygdala |
|---|---|---|---|---|
| Calcarine | 0 | 0 | 0 | 0 |
| DRN | 0 | 0 | 0 | 1 |
| MPFC | 0 | 0 | 0 | 0 |
| Amygdala | 0 | 1 | 0 | 0 |

**DCM.b(:,:,3)**
***[Overload]***

| *from:* / *to:* | Calcarine | DRN | MPFC | Amygdala |
|---|---|---|---|---|
| Calcarine | 0 | 0 | 0 | 0 |
| DRN | 0 | 0 | 0 | 1 |
| MPFC | 0 | 0 | 0 | 0 |
| Amygdala | 0 | 1 | 0 | 0 |

**DCM.c**

| | Boredom | Flow | Overload |
|---|---|---|---|
| Calcarine | 1 | 1 | 1 |
| DRN | 0 | 0 | 0 |
| MPFC | 0 | 0 | 0 |
| Amygdala | 0 | 0 | 0 |

## Model 59 (member of family 6)

**DCM.a**

| from: / to: | Calcarine | DRN | MPFC | Amygdala |
|---|---|---|---|---|
| Calcarine | 1 | 1 | 1 | 1 |
| DRN | 1 | 1 | 1 | 1 |
| MPFC | 1 | 1 | 1 | 1 |
| Amygdala | 1 | 1 | 1 | 1 |

**DCM.b(:,:,1)**
***[Boredom]***

| from: / to: | Calcarine | DRN | MPFC | Amygdala |
|---|---|---|---|---|
| Calcarine | 0 | 0 | 0 | 0 |
| DRN | 0 | 0 | 0 | 0 |
| MPFC | 0 | 0 | 0 | 0 |
| Amygdala | 0 | 1 | 0 | 0 |

**DCM.b(:,:,2)**
***[Flow]***

| from: / to: | Calcarine | DRN | MPFC | Amygdala |
|---|---|---|---|---|
| Calcarine | 0 | 0 | 0 | 0 |
| DRN | 0 | 0 | 0 | 0 |
| MPFC | 0 | 0 | 0 | 0 |
| Amygdala | 0 | 1 | 0 | 0 |

**DCM.b(:,:,3)**
***[Overload]***

| from: / to: | Calcarine | DRN | MPFC | Amygdala |
|---|---|---|---|---|
| Calcarine | 0 | 0 | 0 | 0 |
| DRN | 0 | 0 | 0 | 0 |
| MPFC | 0 | 0 | 0 | 0 |
| Amygdala | 0 | 1 | 0 | 0 |

**DCM.c**

| | Boredom | Flow | Overload |
|---|---|---|---|
| Calcarine | 1 | 1 | 1 |
| DRN | 0 | 0 | 0 |
| MPFC | 0 | 0 | 0 |
| Amygdala | 0 | 0 | 0 |

## Model 60 (member of family 6)

**DCM.a**

| from: / to: | Calcarine | DRN | MPFC | Amygdala |
|---|---|---|---|---|
| Calcarine | 1 | 1 | 1 | 1 |
| DRN | 1 | 1 | 1 | 1 |
| MPFC | 1 | 1 | 1 | 1 |
| Amygdala | 1 | 1 | 1 | 1 |

**DCM.b(:,:,1)**
***[Boredom]***

| from: / to: | Calcarine | DRN | MPFC | Amygdala |
|---|---|---|---|---|
| Calcarine | 0 | 0 | 0 | 0 |
| DRN | 0 | 0 | 0 | 1 |
| MPFC | 0 | 0 | 0 | 0 |
| Amygdala | 0 | 0 | 0 | 0 |

**DCM.b(:,:,2)**
***[Flow]***

| from: / to: | Calcarine | DRN | MPFC | Amygdala |
|---|---|---|---|---|
| Calcarine | 0 | 0 | 0 | 0 |
| DRN | 0 | 0 | 0 | 1 |
| MPFC | 0 | 0 | 0 | 0 |
| Amygdala | 0 | 0 | 0 | 0 |

**DCM.b(:,:,3)**
***[Overload]***

| from: / to: | Calcarine | DRN | MPFC | Amygdala |
|---|---|---|---|---|
| Calcarine | 0 | 0 | 0 | 0 |
| DRN | 0 | 0 | 0 | 1 |
| MPFC | 0 | 0 | 0 | 0 |
| Amygdala | 0 | 0 | 0 | 0 |

**DCM.c**

| | Boredom | Flow | Overload |
|---|---|---|---|
| Calcarine | 1 | 1 | 1 |
| DRN | 0 | 0 | 0 |
| MPFC | 0 | 0 | 0 |
| Amygdala | 0 | 0 | 0 |

## Model 61 (member of family 7)

**DCM.a**

| to: \ from: | Calcarine | DRN | MPFC | Amygdala |
|---|---|---|---|---|
| Calcarine | 1 | 1 | 1 | 1 |
| DRN | 1 | 1 | 1 | 1 |
| MPFC | 1 | 1 | 1 | 1 |
| Amygdala | 1 | 1 | 1 | 1 |

**DCM.b(:,:,1)**
***[Boredom]***

| to: \ from: | Calcarine | DRN | MPFC | Amygdala |
|---|---|---|---|---|
| Calcarine | 0 | 0 | 0 | 0 |
| DRN | 0 | 0 | 0 | 0 |
| MPFC | 0 | 0 | 0 | 1 |
| Amygdala | 0 | 0 | 1 | 0 |

**DCM.b(:,:,2)**
***[Flow]***

| to: \ from: | Calcarine | DRN | MPFC | Amygdala |
|---|---|---|---|---|
| Calcarine | 0 | 0 | 0 | 0 |
| DRN | 0 | 0 | 0 | 0 |
| MPFC | 0 | 0 | 0 | 1 |
| Amygdala | 0 | 0 | 1 | 0 |

**DCM.b(:,:,3)**
***[Overload]***

| to: \ from: | Calcarine | DRN | MPFC | Amygdala |
|---|---|---|---|---|
| Calcarine | 0 | 0 | 0 | 0 |
| DRN | 0 | 0 | 0 | 0 |
| MPFC | 0 | 0 | 0 | 1 |
| Amygdala | 0 | 0 | 1 | 0 |

**DCM.c**

| | Boredom | Flow | Overload |
|---|---|---|---|
| Calcarine | 1 | 1 | 1 |
| DRN | 0 | 0 | 0 |
| MPFC | 0 | 0 | 0 |
| Amygdala | 0 | 0 | 0 |

# Model 62 (member of family 7)

**DCM.a**

| to: \ from: | Calcarine | DRN | MPFC | Amygdala |
|---|---|---|---|---|
| Calcarine | 1 | 1 | 1 | 1 |
| DRN | 1 | 1 | 1 | 1 |
| MPFC | 1 | 1 | 1 | 1 |
| Amygdala | 1 | 1 | 1 | 1 |

**DCM.b(:,:,1)**
***[Boredom]***

| to: \ from: | Calcarine | DRN | MPFC | Amygdala |
|---|---|---|---|---|
| Calcarine | 0 | 0 | 0 | 0 |
| DRN | 0 | 0 | 0 | 0 |
| MPFC | 0 | 0 | 0 | 0 |
| Amygdala | 0 | 0 | 1 | 0 |

**DCM.b(:,:,2)**
***[Flow]***

| to: \ from: | Calcarine | DRN | MPFC | Amygdala |
|---|---|---|---|---|
| Calcarine | 0 | 0 | 0 | 0 |
| DRN | 0 | 0 | 0 | 0 |
| MPFC | 0 | 0 | 0 | 0 |
| Amygdala | 0 | 0 | 1 | 0 |

**DCM.b(:,:,3)**
***[Overload]***

| to: \ from: | Calcarine | DRN | MPFC | Amygdala |
|---|---|---|---|---|
| Calcarine | 0 | 0 | 0 | 0 |
| DRN | 0 | 0 | 0 | 0 |
| MPFC | 0 | 0 | 0 | 0 |
| Amygdala | 0 | 0 | 1 | 0 |

**DCM.c**

| | Boredom | Flow | Overload |
|---|---|---|---|
| Calcarine | 1 | 1 | 1 |
| DRN | 0 | 0 | 0 |
| MPFC | 0 | 0 | 0 |
| Amygdala | 0 | 0 | 0 |

## Model 63 (member of family 7)

**DCM.a**

| to: \ from: | Calcarine | DRN | MPFC | Amygdala |
|---|---|---|---|---|
| Calcarine | 1 | 1 | 1 | 1 |
| DRN | 1 | 1 | 1 | 1 |
| MPFC | 1 | 1 | 1 | 1 |
| Amygdala | 1 | 1 | 1 | 1 |

**DCM.b(:,:,1)**
***[Boredom]***

| to: \ from: | Calcarine | DRN | MPFC | Amygdala |
|---|---|---|---|---|
| Calcarine | 0 | 0 | 0 | 0 |
| DRN | 0 | 0 | 0 | 0 |
| MPFC | 0 | 0 | 0 | 1 |
| Amygdala | 0 | 0 | 0 | 0 |

**DCM.b(:,:,2)**
***[Flow]***

| to: \ from: | Calcarine | DRN | MPFC | Amygdala |
|---|---|---|---|---|
| Calcarine | 0 | 0 | 0 | 0 |
| DRN | 0 | 0 | 0 | 0 |
| MPFC | 0 | 0 | 0 | 1 |
| Amygdala | 0 | 0 | 0 | 0 |

**DCM.b(:,:,3)**
***[Overload]***

| to: \ from: | Calcarine | DRN | MPFC | Amygdala |
|---|---|---|---|---|
| Calcarine | 0 | 0 | 0 | 0 |
| DRN | 0 | 0 | 0 | 0 |
| MPFC | 0 | 0 | 0 | 1 |
| Amygdala | 0 | 0 | 0 | 0 |

**DCM.c**

| | Boredom | Flow | Overload |
|---|---|---|---|
| Calcarine | 1 | 1 | 1 |
| DRN | 0 | 0 | 0 |
| MPFC | 0 | 0 | 0 |
| Amygdala | 0 | 0 | 0 |

## Model 64 (member of family 8)

**DCM.a**

| to: \ from: | Calcarine | DRN | MPFC | Amygdala |
|---|---|---|---|---|
| Calcarine | 1 | 1 | 1 | 1 |
| DRN | 1 | 1 | 1 | 1 |
| MPFC | 1 | 1 | 1 | 1 |
| Amygdala | 1 | 1 | 1 | 1 |

**DCM.b(:,:,1)**
***[Boredom]***

| to: \ from: | Calcarine | DRN | MPFC | Amygdala |
|---|---|---|---|---|
| Calcarine | 0 | 0 | 0 | 0 |
| DRN | 0 | 0 | 0 | 0 |
| MPFC | 0 | 0 | 0 | 0 |
| Amygdala | 0 | 0 | 0 | 0 |

**DCM.b(:,:,2)**
***[Flow]***

| to: \ from: | Calcarine | DRN | MPFC | Amygdala |
|---|---|---|---|---|
| Calcarine | 0 | 0 | 0 | 0 |
| DRN | 0 | 0 | 0 | 0 |
| MPFC | 0 | 0 | 0 | 0 |
| Amygdala | 0 | 0 | 0 | 0 |

**DCM.b(:,:,3)**
***[Overload]***

| to: \ from: | Calcarine | DRN | MPFC | Amygdala |
|---|---|---|---|---|
| Calcarine | 0 | 0 | 0 | 0 |
| DRN | 0 | 0 | 0 | 0 |
| MPFC | 0 | 0 | 0 | 0 |
| Amygdala | 0 | 0 | 0 | 0 |

**DCM.c**

| | Boredom | Flow | Overload |
|---|---|---|---|
| Calcarine | 1 | 1 | 1 |
| DRN | 0 | 0 | 0 |
| MPFC | 0 | 0 | 0 |
| Amygdala | 0 | 0 | 0 |
